# Bioorthogonal Small Molecule Imaging Agents Allow Single-Cell Imaging of MET

**DOI:** 10.1371/journal.pone.0081275

**Published:** 2013-11-12

**Authors:** Eunha Kim, Katherine S. Yang, Ralph Weissleder

**Affiliations:** 1 Center for Systems Biology, Massachusetts General Hospital, Boston, Massachusetts, United States of America; 2 Department of Systems Biology, Harvard Medical School, Boston, Massachusetts, United States of America; Stanford University, United States of America

## Abstract

The hepatocyte growth factor receptor (MET) is a receptor tyrosine kinase (RTK) that has emerged as an important cancer target. Consequently, a number of different inhibitors varying in specificity are currently in clinical development. However, to date, it has been difficult to visualize MET expression, intracellular drug distribution and small molecule MET inhibition. Using a bioorthogonal approach, we have developed two companion imaging drugs based on both mono- and polypharmacological MET inhibitors. We show exquisite drug and target co-localization that can be visualized at single-cell resolution. The developed agents may be useful chemical biology tools to investigate single-cell pharmacokinetics and pharmacodynamics of MET inhibitors.

## Introduction

The most dominant paradigm in drug discovery over the last two decades has been the design of exquisitely selective inhibitors that act on a single target within a disease pathway. However, lack of durable efficacy has challenged this ‘one gene, one drug, one disease’ hypothesis [1]. This is not entirely surprising given the robustness of many biological systems and their ability to utilize redundant networks to overcome inhibition of a single protein [2]. For these reasons, multi-targeting has gained renewed interest and indeed many clinically successful drugs have proven to be less selective than originally thought [3] [4] [5]. This observation, together with a systems understanding of cancer pathways has led to the concept of polypharmacology, i.e. the inhibition of multiple targets within a cell [2]. While combination therapies are an obvious first step towards multi-target inhibition, the deliberate design of a single kinase inhibitor that binds to multiple targets is a newer development [2] [6].

Receptor tyrosine kinases (RTKs) are key regulators of critical cellular processes in mammalian development, cell function and tissue homeostasis [7]. Dysregulation of RTKs has been implicated as causative factors in the development and progression of numerous human cancers [7]. Blockbuster drugs, Gleevec (Bcr-Abl and c-Kit), Herceptin (HER2), and Iressa (EGFR) have spawned intense investigation of other RTKs [8]. One of the emerging kinases of interest is the hepatocyte growth factor receptor (MET), which is widely expressed in epithelial and endothelial cells. MET is a central mediator of cell growth, survival, motility, and morphogenesis during development [9]. Consequently, MET overexpression relative to normal tissue has been detected in various types of cancers [10]. In addition, overexpression of MET is indicative of increased tumor aggressiveness and poor prognosis in cancer patients [11] [12] [13] [14]. A number of different MET inhibitors with varying levels of specificity are currently in clinical trials. These include the monospecific inhibitor, PF04217903, and the broad-spectrum inhibitor, Foretinib (GSK13630898; inhibits MET, AXL, RON, PDGFRα, and KDR) [15]. Despite the growing number of different MET inhibitors and peptide based whole body imaging agents [16], it has been difficult to visualize MET expression, intracellular drug distribution and small molecule MET inhibition. 

It is generally believed that imaging is an invaluable tool in the drug development process. Imaging has been used to better understand the biology and pathophysiology of human cancer, enable earlier diagnosis and allow monitoring of therapeutic drug efficacy. Here we set out to develop a bioorthogonal imaging agent for high resolution imaging in live cells, based on clinical small molecule MET inhibitors. Specifically, we developed a mono-specific MET imaging agent based on PF04217903 [17] and compared its imaging characteristics to an imaging agent based on Foretinib [18], a polypharmacological MET inhibitor in phase III clinical development. Using this technique we were able to perform either very specific MET imaging or single-cell multi-target imaging of different proteins inside living cells. Companion imaging drug (CID) development with mono- and polypharmacologic inhibitors of MET would enable not only specific visualization of MET but also visualization of multiple RTKs at single-cell resolution. Such information can potentially provide new insight for biological understanding of MET and RTKs and, therefore, could help in the development of new drug candidates.

## Materials and Methods

### General experimental procedures

Unless otherwise noted, chemical reactions were carried out under an atmosphere of nitrogen or argon in air-dried glassware with magnetic stirring. Air- and/or moisture-sensitive liquids were transferred via syringe. Organic solutions were concentrated by rotary evaporation at 25 - 60 °C at 15-30 torr. Analytical thin layer chromatography (TLC) was performed using plates cut from glass sheets (silica gel 60 F-254 from Silicycle). Visualization was achieved under a 254 or 365 nm UV light and by immersion in an ethanolic solution of cerium sulfate, followed by treatment with a heat gun. Column chromatography was carried out as “Flash Chromatography” using silica gel G-25 (40-63 μM). 

### Materials

All reagents were obtained from commercial sources and used without further purification. Dry THF, MeOH, DCM, and DMF were obtained from Aldrich (St. Louis, MO). Tz-CFDA [19] and (*E*)-cyclooct-4-enyl 2,5-dioxopyrrolidin-1-yl carbonate (TCO-NHS) [20] were synthesized as described earlier. Histidine-tagged recombinant human MET and AXL, GST-tagged recombinant Human PDGFRα, the z´-LYTE Tyrosine-1,4, and 6 peptide assay kits, ER Tracker Red and Hoechst 33342 were purchased from Invitrogen (Grand Island, NY). GST-tagged recombinant Human KDR and RON were purchased from Promega (Madison, WI). PF04217903 and Foretinib were purchased from Selleck Chemicals (Houston, TX). RIPA buffer and the MET, phospho-MET (Y1234/1235), AXL, and PDGFRα antibodies were from Cell Signaling (Danvers, MA). The RON and KDR antibodies were from AbCam (Cambridge, MA). Recombinant human hepatocyte growth factor (HGF) was from Millipore (Billerica, MA). Odyssey blocking buffer was purchased from LI-COR Biosciences (Lincoln, NE). HALT protease inhibitor cocktail, BCA assay, SuperBlock T20 (TBS) blocking buffer, and SuperSignal West Pico chemiluminescent substrate were from ThermoScientific Pierce (Rockford, IL). 

### Instrumentation

^1^H and ^13^C NMR spectra were recorded at 23°C on a Varian 400 MHz spectrometers. Recorded shifts are reported in parts per million (δ) and calibrated using residual undeuterated solvent. Data are represented as follows: Chemical shift, multiplicity (s = singlet, d = doublet, t = triplet, q = quartet, p = pentet, m = multiplet, br = broad), coupling constant (*J*, Hz) and integration. LC-ESI-MS analysis and HPLC-purifications were performed on a Waters (Milford, MA) LC-MS system. For LC-ESI-MS analyses, a Waters XTerra^®^ C18 5 μm column was used. For preparative runs, an Atlantis^®^ Prep T3 OBDTM 5 μM column was used (eluents 0.1% TFA (v/ v) in water and MeCN; gradient: 0-1.5 min, 5-100% B; 1.5-2.0 min 100% B). z´-LYTE assay fluorescent signal was measured using a Tecan Safire^2^ microplate system (Männedorf, Switzerland). Data were analyzed using Prism 6 (GraphPad, La Jolla, CA) for Mac. Images were collected using a DeltaVision microscope (Applied Precision, Issaquah, WA). 

### Chemical synthesis

NMR-spectra of all the products are available. (File S1)

#### 1-((4-fluorophenyl)carbamoyl)cyclopropanecarboxylic acid (1)

To a solution of cyclopropane-1,1-dicarboxylic acid (2 g, 15.37 mmol) in THF (50 mL), stirred at 0 °C, triethylamine (2143 µL, 15.37 mmol) was added dropwise. The reaction mixture was stirred at 0 °C under nitrogen. 30 minutes later, thionyl chloride (1117 µL, 15.37 mmol) was added dropwise and the reaction mixture was stirred at 0 °C for additional 30 minutes. Then, toward a turbid reaction mixture, a solution of 4-Fluoroaniline (1624 µL, 16.91 mmol) in THF (50 mL) was added dropwise. The reaction mixture was stirred at 0 °C for 1hr. After 1hr stirring, 1N NaOH solution was added. Extraction with Ethyl Acetate for 3 times and combined organic layer was dried over MgSO_4_ and concentrated in vacuo. The resulting brown solid was washed with cold ethyl acetate to give compound **1** (1.94 g, 57%) as a white pinkish solid. ^1^H NMR (400 MHz, Methanol-d4) δ 7.54 (m, 2H), 7.13 – 6.93 (m, 2H), 1.76 – 1.49 (m, 4H); ^13^C NMR (101 MHz,) δ 76.4, 169.6, 162.0, 159.6, 135.6 (d, *J*_C,F_ = 3.0 Hz), 123.3, (d, *J*_C,F_ = 8.1 Hz) 116.3 (d, *J*_C,F_ = 23.2 Hz), 27.6, 20.1; LRMS (ESI): *m/z* calcd for C_11_H_10_FNO_3_ [M-H]^-^ 222.06, found 222.06.

#### *N-*(3-fluoro-4-hydroxyphenyl)-N-(4-fluorophenyl)cyclopropane-1,1-dicarboxamide (2)

To a solution of **1** (1 g, 4.48 mmol) in DMF (8 drop) and THF (2 mL), stirred at 0 °C, a solution of oxalyl chloride (2.24 mL, 4.48 mmol) in DCM (1 mL) was added dropwise. The reaction mixture was stirred at ambient temperature for 2hr. Then, the solution of **1** and oxalyl chloride in DMF and DCM, was added to a solution of 4-Hydroxy-3-fluorophenol (626 mg, 4.93 mmol) and 2,6-Lutidine (519 µL, 4.48 mmol) in THF (2 mL). After additional stirring at 0 °C, the reaction mixture was gradually warmed up to ambient temperature. After completion of the reaction, monitored by TLC and LC-MS, it was quenched by addition of water. Organic material was extracted with ethyl acetate 3 times and the combined organic layer was washed with 1N HCl twice and washed once with NaHCO_3_ (sat). The combined organic layer was dried over MgSO_4_ and concentrated in vacuo. The resulting crude product was diluted with ethyl acetate and a brown solid was obtained by filtration and washed with EA:Hex = 1:3, 1:2 and to 1:1 to give compound **2** (899 mg, 60%). ^1^H NMR (400 MHz, DMSO-d6) δ 10.04 (s, 1H), 9.89 (s, 1H), 9.58 (s, 1H), 7.60 (dd, *J* = 8.9, 5.0 Hz, 2H), 7.51 (dd, *J* = 13.4, 2.5 Hz, 1H), 7.16 – 7.06 (m, 3H), 6.85 (t, *J* = 9.3 Hz, 1H), 1.41 (s, 4H); ^13^C NMR (101 MHz, DMSO-d6) δ 168.1, 167.9, 159.4, 157.1, 151.3, 148.9, 140.9 (d, *J*_C,F_ = 12.1 Hz), 135.2 (d, *J*_C,F_ = 3.0 Hz), 130.8 (d, *J*_C,F_ = 9.1 Hz) 122.3 (d, *J*_C,F_ = 8.1 Hz), 117.0 (dd *J*_C,F_ = 40.4, 3.0 Hz), 115.0 (d, *J*_C,F_ = 22.2 Hz), 109.2 (d, *J*_C,F_ = 23.2 Hz), 31.2, 15.4; LRMS (ESI): *m/z* calcd for C_17_H_14_F_2_N_2_O_3_ [M-H]^-^ 331.20, found 331.12.

#### 1-(4-(benzyloxy)-3-methoxyphenyl)ethanone (3)

A solution of 4-Hydroxy-3-methoxyacetophenone (2 g, 12.04 mmol), benzyl bromide (2.26g, 13.24 mmol) and potassium carbonate (4.99g, 36.11 mmol) in DMF (35mL) was stirred at 45 °C overnight. The next day the reaction mixture was cooled to room temperature and then poured over ice and the resulting solid was obtained by filtration. The resulting solid was washed with water to give compound **3** (3.1 g, quantitative). ^1^H NMR (400 MHz, Chloroform-d) δ 7.54 (d, *J* = 2.0 Hz, 1H), 7.49 (dd, *J* = 8.4, 2.1 Hz, 1H), 7.46 – 7.41 (m, 2H), 7.41 – 7.35 (m, 2H), 7.34 – 7.29 (m, 1H), 6.89 (d, *J* = 8.6 Hz, 1H), 5.23 (s, 2H), 3.94 (s, 3H), 2.54 (s, 3H); ^13^C NMR (101 MHz, Chloroform-d) δ 196.9, 152.5, 149.6, 136.4, 130.8, 128.8, 128.2, 127.3, 123.2, 112.2, 110.7, 70.9, 56.2, 26.3; LRMS (ESI): *m/z* calcd for C_16_H_16_O_3_ [M+H]^+^ 256.11, found 256.13. 

#### 1-(4-(benzyloxy)-5-methoxy-2-nitrophenyl)ethanone (4)

To a solution of **3** (100 mg, 0.39 mmol) in AcOH (1 mL), stirred at 0 °C, a nitric acid solution (300 µL, 63.01 mmol) was added dropwise. The reaction mixture was then warmed up to ambient temperature and stirred at ambient temperature overnight. The next day, the reaction mixture was poured over ice and the resulting solid was obtained by filtration. The resulting solid was washed with water to give compound **4** (71 mg, 60%). ^1^H NMR (400 MHz, Chloroform-d) δ 7.66 (s, 1H), 7.48 – 7.31 (m, 5H), 6.76 (s, 1H), 5.22 (s, 2H), 3.97 (s, 3H), 2.49 (s, 3H); ^13^C NMR (101 MHz) ^13^C NMR (101 MHz, Chloroform-d) δ 200.2, 154.7, 148.7, 138.4, 135.3, 133.2, 129.0, 128.7, 127.7, 108.9, 71.5, 56.8, 30.5, 29.8; LRMS (ESI): *m/z* calcd for C_16_H_15_NO_5_ [M+H]^+^ 302.10, found 302.12.

#### 1-(2-amino-4-(benzyloxy)-5-methoxyphenyl)ethanone (5)

A solution of **4** (1.13 g, 3.76 mmol), iron (841 mg, 15.1 mmol) and ammonium acetate (1.21g, 15.7 mmol) in toluene (1 mL) and water (1 mL) was refluxed overnight. The next day, the reaction mixture was cooled down to ambient temperature and iron was removed by filtration through a celite pad. The resulting filtrate was dissolved with water and extracted with ethylacetate 3 times. The combined organic layer was dried over MgSO_4_ and concentrated in vacuo. Crude product was purified with silica gel column chromatography to give compound **5** (911 mg, 89%) as a yellow solid. ^1^H NMR (400 MHz, Chloroform-d) δ 7.45 – 7.23 (m, 5H), 7.11 (s, 1H), 6.16 (br s, 2H), 6.12 (s, 1H), 5.06 (s, 2H), 3.80 (s, 3H), 2.48 (s, 3H); ^13^C NMR (101 MHz, Chloroform-d) δ 198.4, 154.7, 147.5, 140.2, 136.1, 128.6, 128.1, 127.2, 115.1, 110.7, 100.9, 70.3, 57.2, 27.7; LRMS (ESI): *m/z* calcd for C_16_H_17_NO_3_ [M+H]^+^ 272.12, found 272.20.

#### 7-(benzyloxy)-6-methoxyquinolin-4-ol (6)

A solution of **5** (300 mg, 1.11 mmol) and sodium methoxide (239 mg, 4.42 mmol) in dimethoxyethane was stirred at ambient temperature. After 30 min, ethyl formate (447 µL, 5.53 mmol) was added dropwise. The reaction mixture was stirred at ambient temperature overnight. The next day, the reaction mixture was neutralized with 1N HCl solution. The resulting solid was obtained by filtration and washed with water to give compound **6** (152 mg, 49%) as a brown solid. ^1^H NMR (400 MHz, Methanol-d4) δ 7.85 (d, *J* = 7.2 Hz, 1H), 7.65 (s, 1H), 7.49 (d, *J* = 7.4 Hz, 2H), 7.40 (t, *J* = 7.4 Hz, 2H), 7.35 (d, *J* = 7.3 Hz, 1H), 7.06 (s, 1H), 6.28 (d, *J* = 7.1 Hz, 1H), 5.25 (s, 2H), 3.95 (s, 3H); ^13^C NMR (101 MHz, DMSO-d6) δ 174.6, 152.0, 147.1, 138.4, 136.1, 135.6, 128.5, 128.1, 128.0, 119.3, 107.3, 104.0, 100.6, 70.0, 55.6, 40.2, 39.9, 39.7, 39.5, 39.3, 39.1, 38.9; LRMS (ESI): *m/z* calcd for C_17_H_15_NO_3_ [M-H]^-^ 280.11, found 280.03.

#### 7-(benzyloxy)-6-methoxyquinolin-4-yl trifluoromethanesulfonate (7)

To a solution of **6** (10 mg, 0.04 mmol), DMAP (0.4 mg, 0.004 mmol) and 2,6-lutdine (8 µL, 0.07 mmol) in dichloromethane (0.3 mL), stirred at -20 °C, trifluoromethanesulfonyl chloride (5 µL, 0.05 mmol) was added dropwise. After an additional 5 min stirring at -20 °C, the reaction mixture was gradually warmed up to ambient temperature. 3hr later, the reaction mixture was concentrated in vacuo. Crude product was dissolved with methanol and the resulting brown solid was obtained by filtration and washed with water to give compound **7** (15 mg, quantitative) as a light brown solid. The resulting crude product was used in the next reaction without further purification or characterization. 

#### N-(4-((7-(benzyloxy)-6-methoxyquinolin-4-yl)oxy)-3-fluorophenyl)-*N-*(4-fluorophenyl)cyclopropane-1,1-dicarboxamide (8)

A solution of **2** (78 mg, 0.27 mmol) and **7** (65 mg, 0.20 mmol) in 2,6-lutdine (1 mL) was refluxed for 7 hr. After 7 hr stirring, the reaction mixture was cooled down to ambient temperature and concentrated in vacuo. The crude product was purified with silica gel column chromatography (EA:Hex = 1:1 to 3:1) to give compound **8** (67 mg, 58%) as a white solid. ^1^H NMR (400 MHz, Chloroform-d) δ 10.26 (s, 1H), 8.96 (s, 1H), 8.33 (d, *J* = 5.4 Hz, 1H), 7.66 (dd, *J* = 12.1, 2.4 Hz, 1H), 7.51 (s, 1H), 7.41 – 7.30 (m, 5H), 7.27 (t, *J* = 7.5 Hz, 2H), 7.23 – 7.16 (m, 2H), 7.09 (t, *J* = 8.6 Hz, 1H), 6.91 (t, *J* = 8.6 Hz, 2H), 6.30 (d, *J* = 5.3 Hz, 1H), 5.16 (s, 2H), 1.63 (q, *J* = 4.7, 4.1 Hz, 2H), 1.51 (q, *J* = 5.3, 4.7 Hz, 2H); ^13^C NMR (101 MHz, Chloroform-d) δ 169.9, 168.6, 161.2, 160.2, 158.8, 155.7, 153.2, 152.1, 150.1, 148.8, 146.8, 137.5 (d, *J*_C,F_ = 13.1 Hz), 136.5 (d, *J*_C,F_ = 9.1 Hz), 136.2, 133.0 (d, *J*_C,F_ = 3.0 Hz), 128.8, 128.2, 127.5, 123.8, 123.2 (d, *J*_C,F_ = 7.1 Hz), 116.7 (d, *J*_C,F_ = 3.0 Hz), 115.9 (d, *J*_C,F_ = 23.2 Hz), 110.1 (dd, *J*_C,F_ = 23.2, 3.0 Hz), 109.5 (d, *J*_C,F_ = 2.0 Hz), 102.4 (d, *J*_C,F_ = 6.1 Hz), 99.8 (d, *J*_C,F_ = 4.0 Hz), 70.9 (t, *J*_C,F_ = 4.0 Hz), 56.3 (d, *J*_C,F_ = 3.0 Hz) 29.23, 17.93; LRMS (ESI): *m/z* calcd for C_34_H_27_F_2_N_3_O_5_ [M-H]^-^ 594.19, found 594.26.

#### N-(3-fluoro-4-((7-hydroxy-6-methoxyquinolin-4-yl)oxy)phenyl)-*N-*(4-fluorophenyl)cyclopropane-1,1-dicarboxamide(9)

A solution of **8** (20 mg, 0.03 mmol) and 10 % Pd/C (2 mg) in ethanol (0.3 mL) was stirred at 65 °C for 5 hr under hydrogen. After 5 hr stirring, the reaction mixture was cooled down to ambient temperature and Pd/C was removed by filtration through celite pad and concentrated in vacuo. Crude product was dissolved with ethyl acetate and washed with water. The combined organic layer was dried over MgSO_4_ and concentrated in vacuo to give compound **9** (15 mg, 88%) as a yellow solid. ^1^H NMR (400 MHz, DMSO-d6) δ 9.75 (s, 1H), 9.52 (s, 1H), 9.38 (s, 1H), 7.78 (d, *J* = 5.1 Hz, 1H), 7.27 (dd, *J* = 13.2, 2.4 Hz, 1H), 7.07 – 6.98 (m, 2H), 6.93 – 6.85 (m, 2H), 6.77 (t, *J* = 9.0 Hz, 1H), 6.67 (s, 1H), 6.58 – 6.49 (m, 2H), 5.72 (d, *J* = 5.1 Hz, 1H), 2.71 (s, 2H), 0.85 (t, *J* = 2.9 Hz, 4H); ^13^C NMR (101 MHz, cdcl_3_) δ 171.36, 169.94, 168.64, 161.23, 160.17, 158.79, 155.71, 153.22, 152.08, 150.09, 148.80, 146.76, 137.5 (d, *J*_C,F_ = 13.1 Hz), 136.5 (d, *J*_C,F_ = 9.1 Hz), 136.20, 133.0 (d, *J*_C,F_ = 3.0 Hz), 128.80, 128.24, 127.54, 123.83, 123.2 (d, *J*_C,F_ = 7.1 Hz), 123.12, 116.7 (d, *J*_C,F_ = 3.0 Hz), 115.9 (d, *J*_C,F_ = 23.2 Hz), 110.2 (d, *J*_C,F_ = 3.0 Hz), 110.0 (d, *J*_C,F_ = 3.0 Hz), 109.5 (d, *J*_C,F_ = 2.0 Hz), 102.43, 102.37, 99.8 (d, *J*_C,F_ = 4.0 Hz), 70.86, 60.56, 56.3 (d, *J*_C,F_ = 3.0 Hz), 29.23, 21.17, 17.93, 14.31;LRMS (ESI): *m/z* calcd for C_27_H_21_F_2_N_3_O_5_ [M-H]^-^ 504.14, found 504.18.

#### *tert-*butyl 4-(3-bromopropyl)piperazine-1-carboxylate

A solution of 1-boc-piperazine (100 mg, 0.54 mmol), diisopropylethylamine (187 µL, 1.07 mmol) and 1,3-dibromopropane (164 µL, 1.61 mmol) in 1,4-dioxane (1 mL) was stirred at 90 °C overnight. The next day, the reaction mixture was cooled down to ambient temperature and NaHCO_3_ (sat) was added. Organic material was extracted with ethylacetate 3 times. The combined organic layer was dried over MgSO_4_ and concentrated in vacuo. Crude product was purified with silica gel column chromatography (MeOH : DCM = 1:20 to 1:10) to give compound tert-butyl 4-(3-bromopropyl)piperazine-1-carboxylate (107 mg, 65%) . ^1^H NMR (400 MHz, Chloroform-d) δ 3.42 (t, *J* = 6.6 Hz, 2H), 3.39 – 3.34 (m, 4H), 2.44 (t, *J* = 6.9 Hz, 2H), 2.33 (t, *J* = 5.1 Hz, 4H), 1.97 (p, *J* = 6.7 Hz, 2H), 1.40 (s, 9H); ^13^C NMR (101 MHz, Chloroform-d) δ 154.7, 79.6, 56.4, 53.0, 31.7, 29.9, 28.5; LRMS (ESI): *m/z* calcd for C_12_H_23_BrN_2_O_2_ [M+H]^+^ 307.09, found.

#### tert-butyl 4-(3-((4-(2-fluoro-4-(1-((4-fluorophenyl)carbamoyl)cyclopropanecarboxamido)phenoxy)-6-methoxyquinolin-7-yl)oxy)propyl)piperazine-1-carboxylate (10)

A solution of **9** (15 mg, 0.03 mmol), tert-butyl 4-(3-bromopropyl)piperazine-1-carboxylate (11 mg, 0.04 mmol) and potassium carbonate (15 mg, 0.11 mmol) in DMF (0.5 mL) was stirred at 80 °C for 4 hr. After 4 hr stirring, the reaction mixture was cooled down to ambient temperature and water was added. Organic material was extracted with ethylacetate 3 times and the combined organic layer was dried over MgSO_4_ and concentrated in vacuo. Crude product was purified with silica gel column chromatography (MeOH : DCM = 1:20 to 1:10) to give compound **10** (19 mg, 87%). ^1^H NMR (400 MHz, Chloroform-d) δ 10.11 (s, 1H), 8.48 (s, 1H), 8.39 (d, *J* = 5.4 Hz, 1H), 7.71 (dd, *J* = 12.1, 2.5 Hz, 1H), 7.49 (s, 1H), 7.43 – 7.35 (m, 3H), 7.26 – 7.20 (m, 1H), 7.14 (t, *J* = 8.6 Hz, 1H), 7.03 – 6.94 (m, 2H), 6.34 (dd, *J* = 5.5, 1.1 Hz, 1H), 4.18 (t, *J* = 6.5 Hz, 2H), 3.96 (s, 3H), 3.41 (t, *J* = 5.0 Hz, 4H), 2.57 (t, *J* = 7.2 Hz, 2H), 2.42 (t, *J* = 4.9 Hz, 4H), 2.09 (t, *J* = 6.9 Hz, 2H), 1.77 – 1.68 (m, 2H), 1.62 – 1.53 (m, 2H), 1.39 (s, 9H); ^13^C NMR 170.0, 168.7, 161.2, 160.2, 158.8, 155.7, 154.9, 153.2, 152.4, 150.0, 148.8, 146.9, 137.5 (d, *J*_C,F_ = 12.1 Hz), 136.5 (d, *J*_C,F_ = 9.1 Hz), 133.0 (d, *J*_C,F_ = 3.0 Hz), 123.8, 123.2 (d, *J*_C,F_ = 8.1 Hz), 116.7 (d, *J*_C,F_ = 3.0 Hz), 116.0, 115.8, 115.6, 109.6 (d, *J*_C,F_ = 22.2 Hz), 108.7, 102.3, 99.7, 79.8, 67.3, 56.3 (d, *J*_C,F_ = 3.0 Hz), 55.1, 53.1, 29.2, 28.6, 26.4, 17.9.; LRMS (ESI): *m/z* calcd for C_39_H_43_F_2_N_5_O_7_ [M+H]^+^ 732.31, found 732.32.

#### (E)-cyclooct-4-en-1-yl 4-(3-((4-(2-fluoro-4-(1-((4-fluorophenyl)carbamoyl)cyclopropanecarboxamido)phenoxy)-6-methoxyquinolin-7-yl)oxy)propyl)piperazine-1-carboxylate (11)

To a solution of **10** (3 mg, 0.0045 mmol) and triethylamine (1.25 µL, 0.009 mmol) in DMF (174 µL), a solution of TCO-NHS (1 mg, 0.0037 mmol) was added in DMF (100 µL). The reaction mixture was stirred at ambient temperature for 1 hr was then purified by HPLC to give compound **11** (1.6 mg, 55%) as a white solid. ^1^H NMR (400 MHz, Methanol-d4) δ 8.42 (d, *J* = 5.4 Hz, 1H), 8.29 (s, 2H), 7.83 (dd, *J* = 12.7, 2.4 Hz, 1H), 7.65 (s, 1H), 7.59 – 7.52 (m, 2H), 7.43 (ddd, *J* = 9.0, 2.5, 1.2 Hz, 1H), 7.36 (s, 1H), 7.33 (t, *J* = 8.8 Hz, 1H), 7.12 – 7.02 (m, 2H), 6.50 (dd, *J* = 5.4, 1.1 Hz, 1H), 5.69 – 5.42 (m, 2H), 4.42 – 4.32 (m, 1H), 4.28 (t, *J* = 5.9 Hz, 2H), 3.56 (t, *J* = 5.2 Hz, 4H), 2.89 (t, *J* = 7.4 Hz, 2H), 2.76 (t, *J* = 4.9 Hz, 4H), 2.42 – 2.31 (m, 3H), 2.20 (p, *J* = 6.6 Hz, 2H), 2.06 – 1.88 (m, 4H), 1.79 – 1.65 (m, 3H), 1.65 – 1.63 (m, 4H); LRMS (ESI): *m/z* calcd for C_43_H_47_F_2_N_5_O_7_ [M+H]^+^ 784.34, found 784.35.

#### 5,5-difluoro-7-(3-(4-(3-((4-(2-fluoro-4-(1-((4-fluorophenyl)carbamoyl)cyclopropanecarboxamido)phenoxy)-6-methoxyquinolin-7-yl)oxy)propyl)piperazin-1-yl)-3-oxopropyl)-1,3-dimethyl-5H-dipyrrolo[1,2-c:2',1'-f][*1,3,2*]diazaborinin-4-ium-5-uide (12)

To a solution of **10** (2 mg, 0.0031 mmol) and triethylamine (1.25 µL, 0.009 mmol) in DMF (119 µL), a solution of BODIPY-FL-NHS (1 mg, 0.0026 mmol) in DMF (219 µL) was added. The reaction mixture was stirred at ambient temperature for 1 hr and was purified by HPLC to give compound **12** (1.7 mg, 73.0%) as a yellow solid.^1^H NMR (400 MHz, Methanol-d4) δ 8.42 (d, *J* = 5.4 Hz, 1H), 8.33 (s, 3H), 7.83 (dd, *J* = 12.6, 2.4 Hz, 1H), 7.64 (s, 1H), 7.60 – 7.51 (m, 2H), 7.47 – 7.39 (m, 2H), 7.39 – 7.28 (m, 2H), 7.11 – 6.98 (m, 3H), 6.50 (d, *J* = 5.5 Hz, 1H), 6.33 (d, *J* = 4.0 Hz, 1H), 6.21 (s, 1H), 4.26 (t, *J* = 5.9 Hz, 2H), 4.01 (s, 3H), 3.73 – 3.56 (m, 4H), 3.21 (t, *J* = 7.6 Hz, 2H), 2.87 – 2.73 (m, 4H), 2.63 (dt, *J* = 14.4, 5.0 Hz, 4H), 2.51 (s, 3H), 2.27 (s, 3H), 2.20 – 2.10 (m, 2H), 1.64 (s, 4H); LRMS (ESI): *m/z* calcd for C_48_H_48_BF_4_N_7_O_6_ [M+H]^+^ 906.74, found 906.76.

#### 6-((6-(1-(2-azidoethyl)-1H-pyrazol-4-yl)-1H-[1,2,3]triazolo[4,5-b]pyrazin-1-yl)methyl)quinoline (13)

To a solution of PF-0417903 (50 mg, 0.13 mmol) in DMF (1 mL), stirred at 0 °C, triethylamine (112 µL, 0.81 mmol) was added dropwise. After 5 min, methanesulfonyl chloride (62 µL, 0.81 mmol) was added. The reaction mixture was gradually warmed up to ambient temperature. After 1hr stirring at ambient temperature, sodium azide (157 mg, 2.42 mmol) was added and the reaction mixture was warmed up to 70 °C and stirred for 1hr. The reaction mixture was purified by HPLC to give compound **13** (30 mg, 56%). ^1^H NMR (400 MHz, Chloroform-d) δ 8.65 (s, 1H), 8.44 (dd, *J* = 4.3, 1.7 Hz, 1H), 8.10 (s, 1H), 7.90 (d, *J* = 7.2 Hz, 2H), 7.65 (d, *J* = 8.7 Hz, 1H), 7.62 (d, *J* = 2.0 Hz, 1H), 7.49 (dd, *J* = 8.8, 2.0 Hz, 1H), 7.12 (dd, *J* = 8.3, 4.3 Hz, 1H), 5.76 (s, 2H), 4.10 – 3.91 (m, 2H), 3.43 (dd, *J* = 6.2, 4.8 Hz, 2H); ^13^C NMR (101 MHz, Chloroform-d) δ 150.1, 148.1, 146.7, 138.9, 136.7, 133.0, 130.9, 129.4, 128.6, 127.9, 127.3, 121.4, 119.7, 51.1, 50.2, 50.1; LRMS (ESI): *m/z* calcd for C_19_H_15_N_11_ [M-H+HCO_2_H]^-^ 442.15, found 442.22.

#### 2-(4-(1-(quinolin-6-ylmethyl)-1H-[1,2,3]triazolo[4,5-b]pyrazin-6-yl)-1H-pyrazol-1-yl)ethanamine (14)

A solution of **13** (30 mg, 0.08 mmol) and palladium charcoal (10 mg) in MeOH (0.8 mL) and DCM (0.2 mL) was stirred at ambient temperature under hydrogen atmosphere overnight. The next day, the palladium charcoal was removed by filtration and the filtrate was concentrated in vacuo to give compound **14** (34 mg, quantitative). ^1^H NMR (400 MHz, DMSO-d6) δ 9.21 (s, 1H), 8.88 (dd, *J* = 4.2, 1.7 Hz, 1H), 8.65 (s, 1H), 8.40 – 8.33 (m, 1H), 8.31 (s, 1H), 8.01 (d, *J* = 8.7 Hz, 1H), 7.98 (d, *J* = 1.9 Hz, 1H), 7.81 (dd, *J* = 8.8, 2.1 Hz, 1H), 7.52 (dd, *J* = 8.3, 4.2 Hz, 1H), 6.14 (s, 2H), 4.17 (t, *J* = 6.2 Hz, 2H), 2.98 (t, *J* = 6.2 Hz, 2H);^13^C NMR (101 MHz, DMSO-d6) δ 151.4, 148.8, 147.7, 147.3, 142.5, 139.0, 138.2, 136.6, 133.9, 131.8, 130.0, 129.9, 128.1, 127.7, 122.4, 119.4, 55.6, 50.5, 42.3; LRMS (ESI): *m/z* calcd for C_19_H_15_N_11_ [M-H+HCO_2_H]^-^ 416.16, found 416.69.

#### (E)-cyclooct-4-en-1-yl (2-(4-(1-(quinolin-6-ylmethyl)-1H-[1,2,3]triazolo[4,5-b]pyrazin-6-yl)-1H-pyrazol-1-yl)ethyl)carbamate (15)

A solution of **14** (5 mg, 0.01 mmol), TEA (2 µL, 0.02 mmol) and TCO-NHS (4 mg, 0.02 mmol) in DMF (0.2 mL) was stirred at ambient temperature for 1 hr. The reaction mixture was purified by HPLC to give compound **15** (3.1 mg, 44%).

^1^H NMR (400 MHz, Deuterium Oxide) δ 9.02 (s, 1H), 8.79 (dd, *J* = 4.3, 1.7 Hz, 1H), 8.39 (s, 1H), 8.32 (dd, *J* = 8.3, 1.7 Hz, 1H), 8.22 (d, *J* = 0.7 Hz, 1H), 7.98 (d, *J* = 8.5 Hz, 2H), 7.87 – 7.80 (m, 1H), 7.52 – 7.44 (m, 1H), 6.12 (s, 2H), 5.39 – 5.17 (m, 2H), 4.24 (t, *J* = 5.7 Hz, 2H), 4.14 – 4.03 (m, 1H), 3.48 (td, *J* = 5.9, 2.9 Hz, 2H), 2.13 – 1.99 (m, 2H), 1.75 – 1.40 (m, 6H), 1.34 – 1.22 (m, 2H); LRMS (ESI): *m/z* calcd for C_28_H_29_N_9_O_2_ [M+H]^+^ 523.24, found 523.25.

### Kinase assays

The IC_50_ of Foretinib, Foretinib-TCO (11), Foretinib-BODIPY-FL (12), PF04217903, and PF04217903-TCO (15) were determined using the z´-LYTE assay kit. The Tyr6 peptide kit was used to measure MET, RON, and AXL activity. The Tyr4 peptide kit was used for PDGFRα and the Tyr1 peptide kit was used to measure KDR activity. All assays were run in accordance with the manufacturer’s instructions, with minor modification. Briefly, MET, RON, and KDR were used at 0.2 μg/ml, 0.8 μg/ml, and 0.7 μg/ml, respectively. The kinase reaction buffer for MET, RON, and KDR was supplemented with 0.67% DMSO. AXL was used at 1.5 μg/ml and the reaction buffer was supplemented with 0.01% sodium azide and 0.67% DMSO. PDGFRα was used at 2.5 μg/ml and the reaction buffer was supplemented with 1mM DTT, 2mM MnCl_2_, and 0.67% DMSO. Inhibitors were prepared by 4-fold serial dilution at 75X the final concentration in 100% DMSO (8 μM to 0.1 nM). This stock was then diluted to 3X the final concentration in kinase reaction buffer. 5 μl of the 3X stock was added to the final 15 μl reaction volume. Both the z´-LYTE control phosho-peptide and the z´-LYTE peptide were used at a final concentration of 2 μM. ATP was added at a final concentration of 50 μM for MET and AXL, 10 μM for RON and PDGFRα and 75 μM for KDR to initiate the reaction, which proceeded for 1 hour at room temperature. Development reagent was then incubated for 1 hour at room temperature. After terminating the reaction with stop reagent, the coumarin and FRET based fluorescein emission was measured on a TECAN Saffire^2^ plate reader (ex: 400 nm, em: 445 and 520 nm). IC_50_ values were obtained by fitting the dose-response curves using Prism 6 (GraphPad).

### Cell lines

HT-29, SK-BR-3, MDA-MB-436, MDA-MB-231, HCC1937, HCC1395, and HCC38 cells were from ATCC (Manassas, VA). OVCA429 [21] and A2780 [22] cells were kindly provided by Dr. Michael Birrer (Massachusetts General Hospital, Boston, MA). HT-29 and SK-BR-3 cells were maintained in Dulbecco’s Modified Eagle Medium supplemented with 10% fetal bovine serum, 100 I.U. penicillin, 100 μg/ml streptomycin, and 2 mM L-glutamine. All other cell lines were maintained in RPMI 1640 supplemented with 10% fetal bovine serum, 100 I.U. penicillin, 100 μg/ml streptomycin, and 2 mM L-glutamine

### Live cell fluorescence microscopic imaging of Foretinib-TCO and Foretinib-BODIPY-FL

OVCA429 cells were plated at 5000 cells per well in 96-well black μ-clear bottom plates (Grenier Bio-One) and were grown for 48-72 hrs. On the day of imaging, cells were incubated with a final concentration of 40, 200 and 1000 nM (0.1% DMSO in growth media) of Foretinib-TCO (11) and Foretinib-BODIPY-FL (12) for 30 min at 37°C. Cells were washed three times with media (5 min each). In the case of Foretinib-TCO, cells were then incubated with 1 μM Tz-CFDA (0.1% DMSO in growth media) for 30 min at 37°C. After washing with media several times over 2 hrs, live cells were imaged in a humidified environmental chamber of a DeltaVision microscope using a 40X objective. 

### Fixed cell fluorescence microscopic imaging of PF04217903-TCO and Foretinib-TCO with Immunostaining of MET.

OVCA429 cells were plated at 5000 cells per well in 96-well black μ-clear bottom plates (Grenier Bio-One) and were grown for 48-72 hrs. On the day of imaging, cells were incubated with a final concentration of 40 nM (0.1% DMSO in growth media) of PF04217903 (15) and 200 nM (0.1% DMSO in growth media) of Foretinib-TCO (11) for 30 min at 37°C. Cells were washed three times with media (5 min each) and then incubated with 1 μM Tz-CFDA (0.1% DMSO in growth media) for 30 min at 37°C. Cells were washed with media several times over 2 hrs and fixed in 2% paraformaldehyde (in PBS) for 10 min at room temperature. Following 3 washes, 5 min each, with PBST (PBS with 0.1% Tween-20), cells were permeabilized with 100% MeOH for 10 min at room temperature. Cells were blocked in Odyssey Blocking Buffer (LI-COR Biosciences) for 1 hr at room temperature. Primary antibody staining with a MET (1:3000, Cell Signaling) was done overnight at 4°C in Odyssey Blocking Buffer. Following three washes (5 min each) with PBST, cells were stained with a donkey α-rabbit Alexa Fluor 647 conjugated secondary antibody (1:200, Invitrogen) for 1 hour at room temperature. Cells were washed once with PBST and twice with PBS. Cell nuclei were stained with Hoechst 33342 (Invitrogen). Imaging was done on a DeltaVision microscope (Applied Precision) using a 40X objective.

### Fixed cell fluorescence microscopic imaging of Foretinib-TCO/Tz-CFDA with Immunostaining of 5 different RTKs

OVCA429 cells were plated at 5000 cells per well in 96-well black μ-clear bottom plate (Grenier Bio-One, Monroe, NC) and were grown for 48-72 hrs. On the day of imaging, cells were incubated with a final concentration of 200 nM (0.1% DMSO in growth media) of Foretinib-TCO (11) for 30 min at 37°C. Cells were washed three times with media and were then incubated with 1 μM Tz-CFDA (0.1% DMSO in growth media) for 30 min at 37°C. Cells were washed with media several times over 2 hrs and fixed in 2% paraformaldehyde (in PBS) for 10 min at room temperature. Following 3 washes, 5 min each, with PBST (PBS with 0.1% Tween-20), cells were permeabilized with 100% MeOH for 10 min at room temperature. Cells were blocked in Odyssey Blocking Buffer (LI-COR Biosciences) for 1 hr at room temperature. Primary antibody staining with a MET (1:3000, Cell Signaling), PDGFRα (1:1000, Cell Signaling), RON (1:50, Abcam), AXL (1:100, Cell Signaling), or KDR (1:1000, Abcam) antibody was done overnight at 4°C in Odyssey Blocking Buffer. Following three washes (5 min each) with PBST, cells were stained with a donkey α-rabbit Alexa Fluor 647 conjugated secondary antibody (1:200, Invitrogen) for 1 hour at room temperature. Cells were washed once with PBST and twice with PBS. Cell nuclei were stained with Hoechst 33342 (Invitrogen). Imaging was done on a DeltaVision microscope (Applied Precision) using a 40X objective. 

### Live cell fluorescence microscopic imaging of PF04217903-TCO and Foretinib-TCO

OVCA429 cells were plated at 5000 cells per well in 96-well black μ-clear bottom plates (Grenier Bio-One) and were grown for 48-72 hrs. On the day of imaging, cells were incubated with a final concentration of 40 nM (0.1% DMSO in growth media) of PF04217903 (15) and 200 nM (0.1% DMSO in growth media) of Foretinib-TCO (11) for 30 min at 37°C. Cells were washed three times with media (5 min each) and then incubated with 1 μM Tz-CFDA (0.1% DMSO in growth media) for 30 min at 37°C. After washing with media several times over 2 hrs, live cells were imaged in a humidified environmental chamber of a DeltaVision microscope using a 40X objective. 

### Western blot

OVCA429, SK-BR-3, A2780, MDA-MB-436, MDA-MB-231, HCC1937, HCC1395, and HCC38 cells were plated in one 35mm well each. 48 hrs after plating, the cells were washed twice with ice-cold PBS, and scraped in RIPA buffer (Cell Signaling Technology) containing 1X HALT protease inhibitor cocktail (ThermoScientific Pierce). Lysates were passed through a 23g syringe 5 times, sonicated for 40 sec, and vortexed every min for 5 min to solubilize the proteins. Lysates were then centrifuged for 15 min at 14,000 x g (4°C). Total protein from the supernatant was determined using the BCA assay (ThermoScientific Pierce). Equal amounts of protein from each cell sample was boiled for 5 min and then run on a 4-12% Bis-Tris Novex NuPAGE gel (Invitrogen) for 1 hr at 200V. Protein was transferred to nitrocellulose, which was blocked using SuperBlock T20 (TBS) buffer. Blots were briefly washed in TBST (TBS with 0.1% Tween-20). Primary antibodies against MET (1:2000), RON (1:5000), KDR (1:1000), AXL (1:1000), or PDGFRα (1:1000) were diluted in 10% SuperBlock/TBST and incubated overnight at 4°C. Blots were washed three times, 5 min each, in TBST and then incubated in goat α-rabbit IgG HRP conjugated secondary antibody diluted in 10% SuperBlock/TBST. Blots were again washed three times, 5 min each, in TBST and were detected using SuperSignal West Pico chemiluminescent substrate (ThermoScientific Pierce). Blots for MET, RON, and PDGFRα were from blots that were stripped using Restore^TM^ western blot stripping buffer (ThermoScientific).

For analysis of MET phosphorylation following Foretinib inhibition, OVCA429 cells were plated in 6-well plates 48 hrs before the assay. Cells were serum-starved in media containing 0.1% fetal bovine serum ~16 hrs before the assay. On the day of the assay, 0, 0.1, 0.5, or 1 μM Foretinib, Foretinib-TCO (11), or Foretinib-BODIPY-FL (12) were diluted in media containing 1 mg/ml BSA. Cells were incubated with Foretinib or its derivatives for 1 hr at 37°C. Phosphorylation was then stimulated with 100 ng/ml HGF diluted in RPMI with 1 mg/ml BSA for 5 min at 37°C. Cell lysates were then prepared and run on a gel as described above. Western blot analysis was performed as described above using the MET and phospho-MET primary antibodies (1:1000). Total MET and phospho-MET expression was quantified using densitometry (ImageJ).

## Results

### Molecular design of mono- and poly-pharmacologic agents

**Figure 1:**
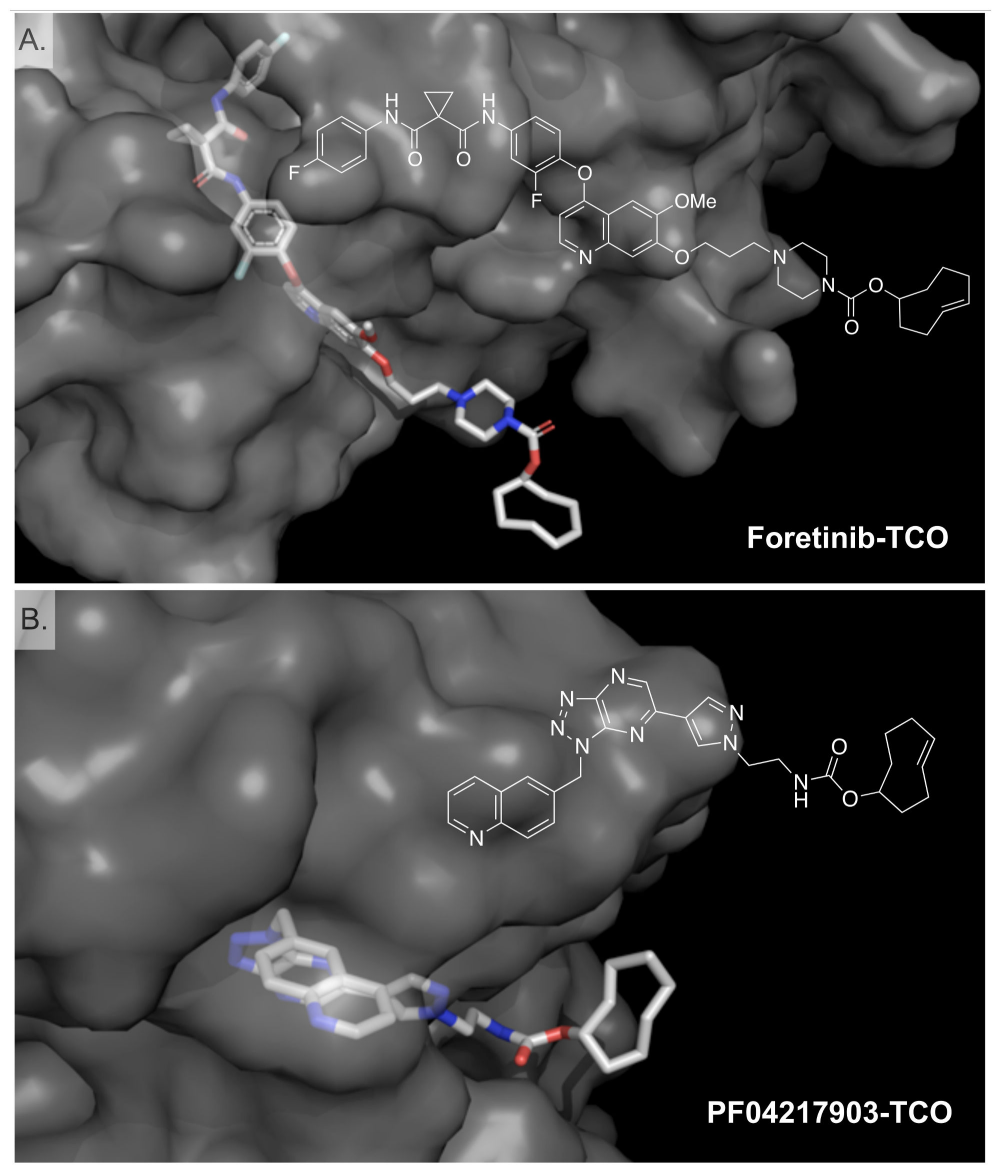
Crystal structure of MET (gray) showing the design of the bioorthogonal MET imaging agents. (A) Foretinib-TCO (B) PF04217903-TCO. Note that the bioorthogonal transcyclooctene (TCO) is predicted to project outside from the target so that it is available for reaction with the fluorescent counter partner. (PDB ID: 3LQ8 and 3ZXZ) 3D models were rendered using PyMol.

**Figure 2:**
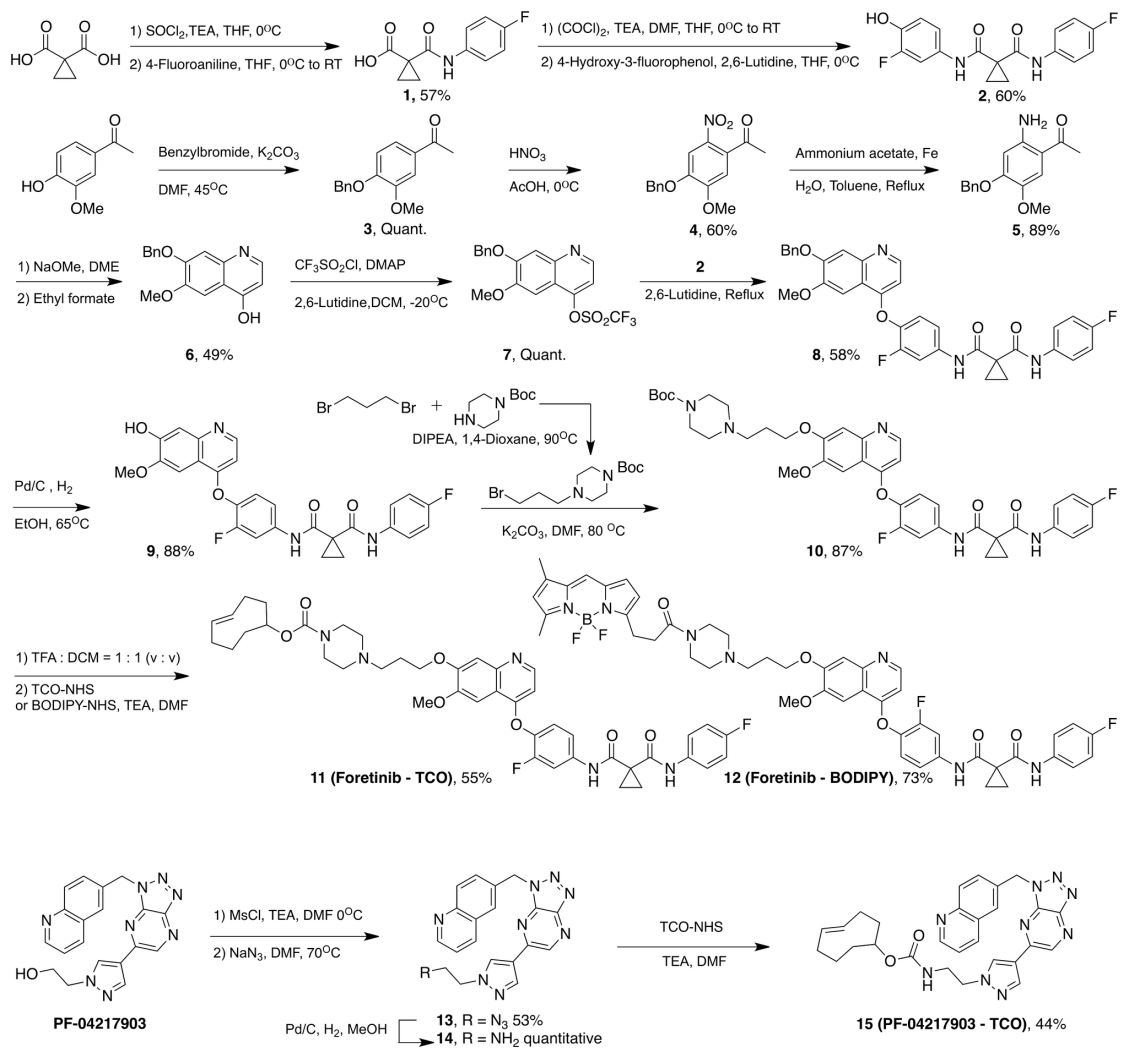
Synthetic scheme of Foretinib-TCO (11), Foretinib-BODIPY-FL (12) and PF04217903-TCO (15). Boc = *tert*-butyloxycarbonyl; Bn = Benzyl; BODIPY-FL = 4,4-difluoro-5,7-dimethyl-4-bora-3a,4a-diaza-s-indacene-3-propionyl; DMAP = 4-dimethylaminopyridine; DCM = dichloromethane; DIPEA = N,N-Diisopropylethylamine; DME = dimethoxyethane; DMF = dimethylformamide; Ms = methanesulfonyl; TCO = trans-cyclooctene; TEA = triethylamine; TFA = trifluoroacetic acid; THF = tetrahydrofuran; NHS = *N*-hydroxysuccinimide.

We have developed a polypharmacologic companion imaging drug (PCID) using the multi-targeting MET inhibitor, *N1*’-[3-fluoro-4-[[6-methoxy-7-(3-morpholinopropoxy)-4-quinolinyl]oxy]phenyl]-*N1*-(4-fluorophenyl)cyclopropane-1,1-dicarboxamide (Foretinib) as a chemical scaffold (Figure 1A) and a specific MET CID based on the selective MET inhibitor, 2-(4-(3-(quinolin-6-ylmethyl)-3*H*-[1,2,3]triazolo[4,5-*b*]pyrazin-5-yl)-1*H*-pyrazol-1-yl)ethanol (PF04217903) (Figure 1B). Crystal structure analysis indicated that the morpholine residue of Foretinib is not involved in ligand-protein interaction, is exposed to the solvent and could therefore be available for modification with trans-cyclooctene (TCO) (Figure S1). An amine reactive Foretinib derivative was thus synthesized, which could act as the starting material for different imaging agents, as described previously with minor modification (Figure 2). Briefly, chlorination of cyclopropane-1,1-dicarboxylic acid with thionyl chloride, followed by reaction with 4-fluoroaniline, resulted in compound **1**. Repeating chlorination of **1** with oxalylchloride and addition of 4-hydroxy-3-fluorophenol yielded compound **2**. Benzyl protection of 4-hydroxy-3-methoxy acetophenone afforded compound **3** and nitration of **3** with nitric acid in acetic acid resulted in compound **4**. Reduction of **4** with iron and ammonium acetate in refluxed toluene and water resulted in compound **5**. Cyclization of compound **5** with ethylformate in basic condition resulted in **6**, and triflation of compound **6** resulted in compound **7**. Coupling of compound **2** and **7** in refluxed 2,6-lutidine afforded compound **8** and benzyl deprotection by hydrogenation with palladium charcoal gave compound **9**. Coupling of **9** with piperazine derivatives (prepared by reaction between 1,3-dibromopropane and *N*-Boc-piperazine) in basic condition resulted in compound **10**. Finally, the Boc group was deprotected with TFA and the resulting secondary amine was recovered. The selective MET intermediate scaffold based on PF04217903 was synthesized similarly [15] in 3 steps (overall yield of 23%; Figure 2 and Figure S2A and B).

### Characterization of companion imaging drugs

**Figure 3:**
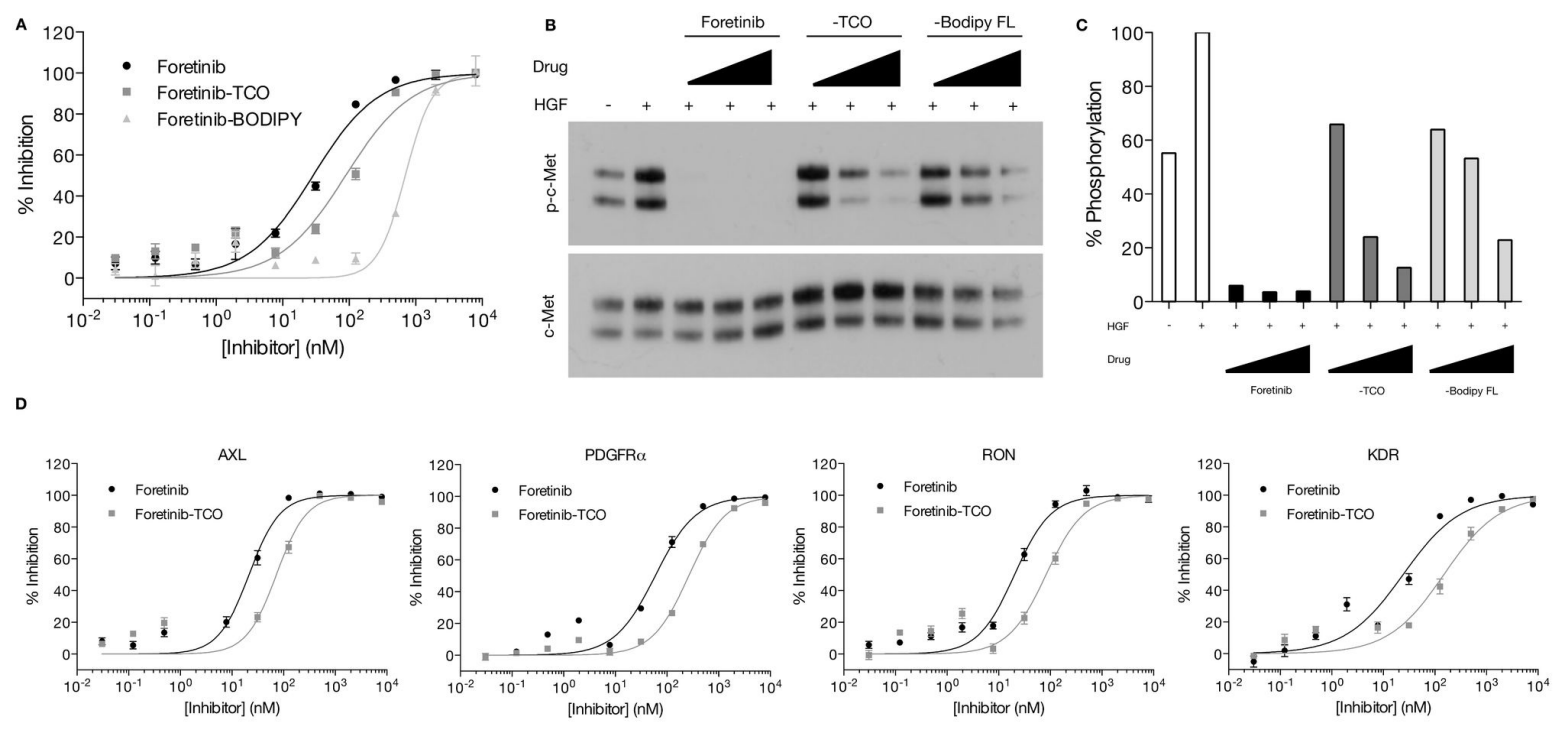
Inhibitory effect of Foretinib based imaging agents. (A) The IC_50_ values for Foretinib, Foretinib-TCO (**11**) and Foretinib-BODIPY-FL (**12**) against purified MET were determined using the z’-lyte kinase assay. Note the much lower affinity of the fluorochrome conjugated drug compared to the bioorthogonal version. (B) Representative western blot of MET phosphorylation inhibition by Foretinib, Foretinib-TCO (**11**) and Foretinib-BODIPY-FL(**12**) in OVCA429 cells. Following pre-treatment with increasing concentrations of inhibitors (0, 100, 500, or 1000 nM, respectively), MET phosphorylation was stimulated with HGF for 10 min, followed by cell lysis, SDS-PAGE, and Western blot with MET and phospho-MET antibodies. (C) Densiometric quantification of MET phosphorylation from the Western blot data using ImageJ. (D) The IC_50_ values for Foretinib and Foretinib-TCO (**11**) against purified AXL, PDGFRα, RON and KDR were determined using the z’-lyte kinase assay. R^2^ values for the dose-response curve fit (GraphPad, Prism) were 0.92 or greater.

To convert the above amine containing intermediates into different imaging agents, we pursued two approaches: direct conjugation to a fluorochrome (BODIPY-FL) and two-step bioorthogonal clickable reaction via TCO-drug and tetrazine(Tz)-BODIPY FL. The inhibitory effects of Foretinib, Foretinib-TCO (**11**) and Foretinib-BODIPY-FL (**12**) were evaluated using a FRET based MET kinase activity assay. Direct modification of Foretinib with the BODIPY-FL fluorochrome (12) led to a significant loss in drug activity, decreasing the IC_50_ over 24-fold (from 28.7 nM [23] to 697.4 nM; Figure 3A and Table 1). Interestingly, the smaller TCO modification only modestly impacted the IC_50_ value of Foretinib (88.6 nM; Figure 3A and Table 1). To further assess the impact of the BODIPY-FL (12) and the TCO (**11**) modifications on Foretinib activity, Western blot experiments were done for MET phosphorylation. OVCA429 ovarian cancer cells were pre-treated with increasing concentrations of Foretinib, Foretinib-BODIPY-FL (**12**) or Foretinib-TCO (**11**). Cells were subsequently lysed and Western blot analysis was done for phosphorylated MET, as well as total MET expression (Figure 3B and C). MET exhibited some basal phosphorylation, consistent with the high overexpression of MET in OVCA429 cells and with previous reports [24]. Stimulation with HGF led to a significant increase in MET phosphorylation. For total MET expression, we observed two different MET protein bands, the 145 kDa beta-chain and the unprocessed 170 kDa precursor [25] [26] [27]. At all concentrations tested, Foretinib treatment led to a complete decrease in MET phosphorylation. Both Foretinib-TCO (**11**) and Foretinib-BODIPY-FL (**12**) pre-treatment led to a decrease in MET phosphorylation, with the TCO (**11**) modification exhibiting greater potency than the BODIPY-FL (**12**) version (Figure 3B and C). This trend is consistent with the assay using purified MET, but the absolute inhibitory effect may be different in cell culture due to the abundance of other targets available for interaction with Foretinib and its derivatives. Similarly, TCO modification of PF04217903 (**15**) showed minimal reduction of the IC_50_ value from 0.54 nM to 1.48 nM (Figure S2C). Figure S3 compares imaging of Foretinib-TCO to Foretinib-BODIPY-FL in OVCA429 ovarian cancer cells. The former showed a dose-dependent increase in cellular fluorescence signal (Figure S3) whereas the latter shows cellular staining only at the highest concentration tested (in addition to or because of its poor target binding). Collectively, therefore, we confirmed the efficiency of the two-step bioorthogonal imaging strategy for PCID. Focusing on the two-step bioorthogonal imaging strategy we next assessed the affinity of Foretinib-TCO against other target kinases: AXL, RON, PDGFRα, or KDR. Similar to the results obtained using purified MET, the TCO (**11**) modification only minimally impacted the IC_50_ value for each of the protein targets tested (Figure 3D and Table 1). On average, TCO modification still resulted in an affinity ligand in the low nM range (Table 1) suggesting its use as a model PCID. 

**Table 1:** IC_50_ values of Foretinib, Foretinib-TCO (11), and Foretinib-BODIPY-FL (12) for the indicated recombinant human kinases.

**Compound**	**MET**	**AXL**	**RON**	**PDGFRα**	**KDR**
Foretinib	28.7	21.4	20.2	59.2	24.3
Foretinib-TCO	88.6	72.5	82.7	264.6	149.9
Foretinib-BODIPY-FL	697.4	ND	ND	ND	ND
Fold Change (TCO/Foretinib)	3.1	3.4	4.1	4.5	6.2

All IC_50_ values are in nM and R^2^ values for the dose-response curve fit (GraphPad, Prism) were 0.92 or greater. Fold change is Foretinib-TCO (**11**) IC_50_ value divided by Foretinib IC_50_ value. ND: not determined.

### Single-cell distribution of mono- and poly-pharmacologic companion imaging drugs

**Figure 4:**
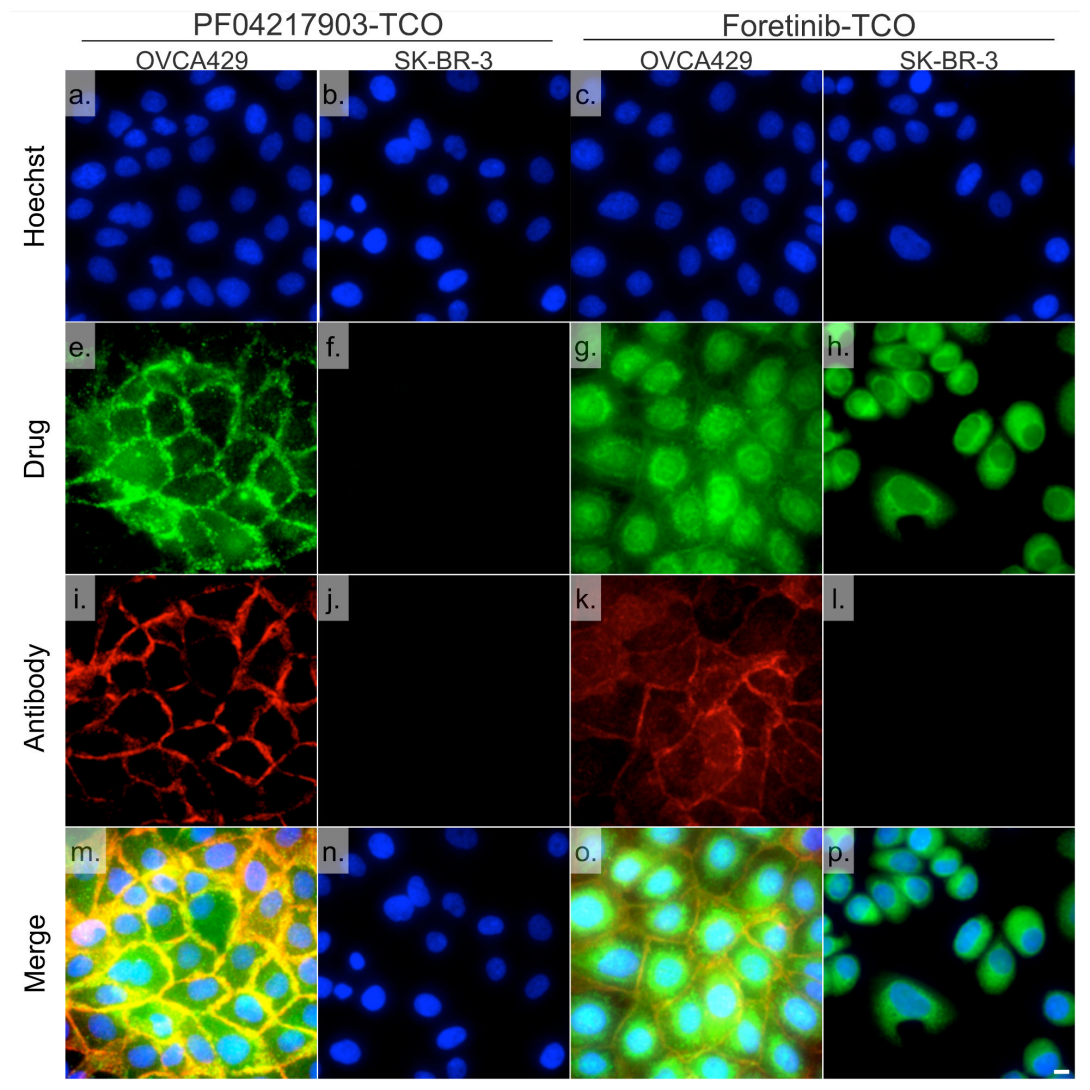
Bioorthogonal labeling using the specific MET CID PF04217903-TCO (15) or the PCID Foretinib-TCO (11) in OVCA429 (MET positive) and SK-BR-3 (MET negative) cells. Cells were incubated for 1hr with 200 nM Foretinib-TCO (**11**) or 40 nM PF04217903-TCO (**15**), washed and incubated for 30 min with 1 μM Tz-CFDA (e-h) for bioorthogonal reaction inside living cells. After fixation with 2% paraformaldehyde, MET was labeled using a MET primary antibody and AlexaFluor 647 labeled secondary antibody (i-l). After nuclear staining with Hoechst 33342 (a-d) for 10 min, 40X images were collected using a DeltaVision microscope. Note the excellent co-localization between the MET antibody and affinity ligands on the membrane of the cells (m-p). Scale bar: 10 μm.

**Figure 5:**
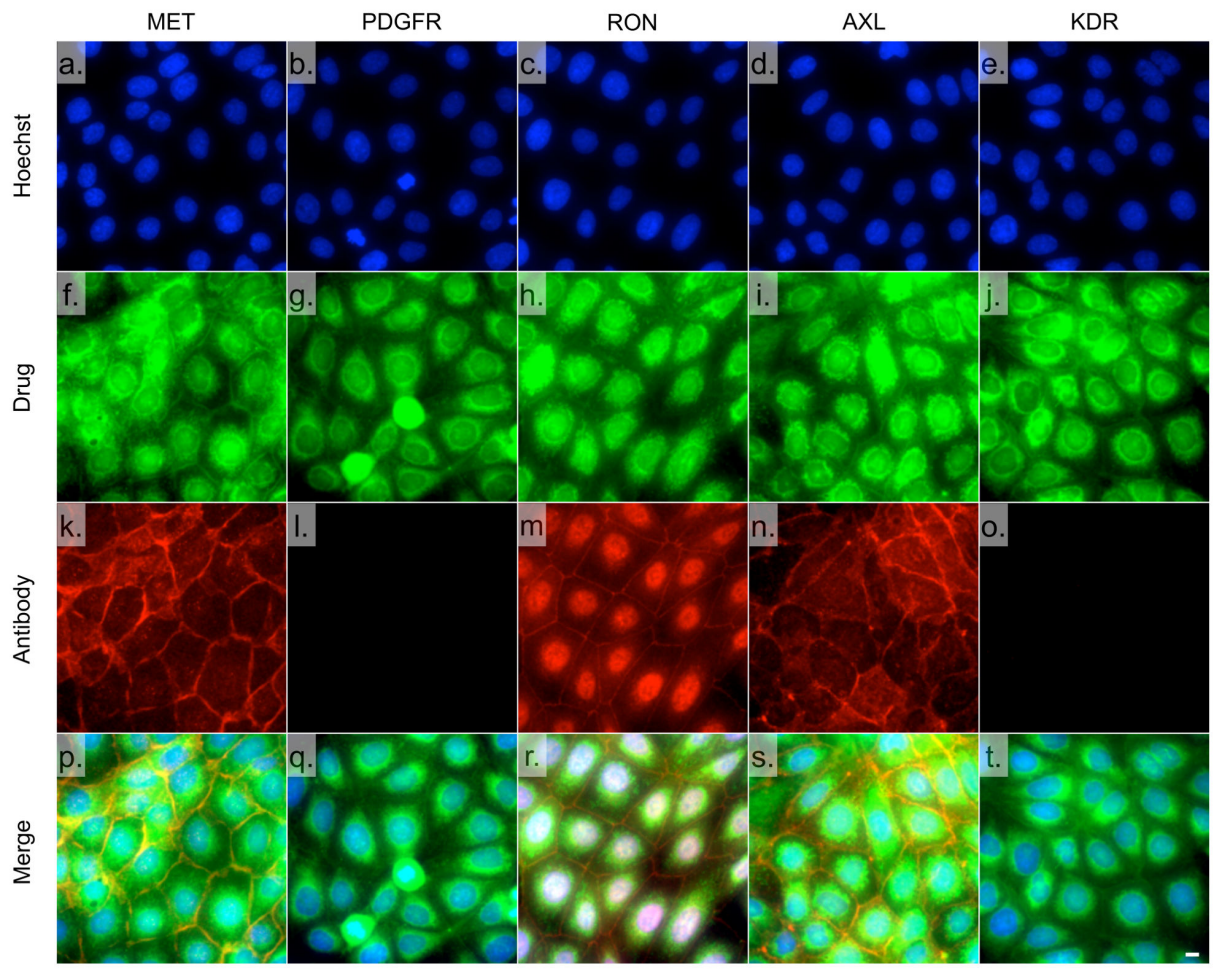
OVCA429 cells were incubated for 30 min with 200 nM Foretinib-TCO (11), washed, and incubated for 30 min with 1 μM Tz-CFDA (f-j, green) for bioorthogonal reaction inside living cells. Cells were subsequently fixed and stained with antibodies (red) for MET (a,f,k,p), PDGFRα (b,g,l,q), RON (c,h,m,r), AXL (d,i,n,s), and KDR (e,j,o,t). Cell nuclei were stained with Hoechst 33342 (blue, a-e) for 10 min and 40x images were collected using a DeltaVision microscope. Note that the PCID allows imaging of multiple targets. Scale bar: 10 μm.

Given the affinity and cellular uptake of the bioorthogonal agents we next determined target localization by fluorescence microscopy. We chose carboxyfluorescein diactate-tetrazine (Tz-CFDA) to reveal Foretinib-TCO (**11**) given prior results in live cell imaging [15]. These experiments were performed in OVCA429 ovarian cancer cells because of their high MET expression as determined by Western blot (Figure S4). We first tested PF04217903-TCO (**15**), given its exquisite affinity and specificity for MET. Compared to antibody co-staining for MET, there was superb co-localization of the drug and its target (Figure 4 and Figure S5, Manders correlation coefficient of colocalization test about selected image was 0.969). A control experiment with unlabeled Foretinib/Tz-CFDA confirmed the low background signal as well as specificity of the TCO/Tz based two-step bioorthogonal labeling technique (Figure S5). When SK-BR-3 cells that lack MET were used for the same imaging experiments, PF04217903-TCO (**15**) showed no significant staining compared to Foretinib-TCO (**11**) (Figure 4). Interestingly, Foretinib-TCO showed a different pattern. In addition to some membrane staining, there was also significant other cellular staining within the cell, presumably the cytoplasm/perinuclear region (Figure 4). Similar staining patterns were also observed by live cell imaging for both PF04217903-TCO and Foretinib-TCO (Figure S6). We next investigated the phenotype of intracellular Foretinib-TCO distribution (Figure 5). We wanted to better understand the poly-pharmacologic drug distribution to understand where the drug localizes. We were specifically interested in determining whether the observed cellular staining co-localized with any of the other known Foretinib targets. To further probe the polypharmacologic distribution of Foretinib, we used correlative antibody staining against AXL, RON, PDGFRα, KDR, and MET. Figure 5 summarizes these experiments showing co-localization of Foretinib-TCO (**11**) with some of the other known targets. Consistent with the enzymatic assay result, Foretinib-TCO (**11**)/Tz-CFDA shows good co-localization with the membrane signal from the MET, AXL, and RON antibody staining (Figure 5). In addition the red fluorescent nuclear signal from immunostaining with the RON antibody [28] shows good co-localization with Foretinib-TCO (**11**)/Tz-CFDA staining. There was less co-localization with PDGFRα and KDR, likely due to the low expression of these proteins in OVCA429 cells (Figure S4). Overall, we observed very good co-localization between the specific MET inhibitor (PF04217903-TCO) and MET. In contrast, Foretinib-TCO/Tz-CFDA staining not only appears in the membrane but also in the cytoplasm as well as the nucleus. Based on recent kinome studies [29], these patterns are most likely explained by the very broad inhibition of > 30 kinases throughout the cell by this compound. Therefore, our results shows that the Foretinib-TCO (**11**)/Tz-CFDA bioorthogonal two-step labeling procedure is useful for imaging polypharmacologic distribution of drug targets not only in fixed cells, but also in live cells. Moreover PCID, combining with the image based phenotypic screening system, will be a useful chemical tool for new polypharmacologic drug discovery.

## Discussion

The ability of many drugs, often unintended, to interact with multiple proteins is commonly referred to as polypharmacology. Using Foretinib as a model system we show that bioorthogonal conjugates of the drug can be used to image drug binding to its multiple targets. Specifically, we i) investigated the IC_50_ for most known binding partners and showed them to be similar to that of the parent drug, ii) showed that the drug distribution inside single cells can be visualized (important in confirming target localization) and iii) that cellular images reflect the distribution and abundance of multiple drug targets (as shown by Western and comparative selective MET imaging). 

 The approach shown here has two practical and potentially far reaching applications: i) synthesis of imageable drug analogs to study their distribution in vivo and ii) to develop new multi-target imaging agents that can be translated to whole body and clinical imaging. While we used a bioorthogonal two-step procedure for cellular imaging to optimize spatial resolution we anticipate that the development of small footprint fluorochromes will ultimately enable in vivo imaging at the whole body level [30]. This will be further facilitated by the concurrent development of red-shifted cell permeable fluorochromes [31], turn-on fluorochromes [32], fluorochromes with improved pharmacokinetics and fluorochromes that can be labeled with radioisotopes for PET imaging [33].

## Supporting Information

Figure S1 **Molecular design of polypharmacology companion imaging drugs (PCID) based on the crystal structures of Foretinib.** a) Crystal structure of MET in complex with Foretinib (PDB ID: 3LQ8). b) Crystal structure prediction of MET in complex with Foretinib-TCO. 3D models were rendered using PyMol.
(TIFF)

Figure S2 **Molecular analysis of MET-specific imaging drugs based on the PF04217903 scaffold.** a) Crystal structure of MET in complex with PF04217903 (PDB ID: 3zxz). b) Crystal structure prediction of MET in complex with PF04217903-TCO. 3D models were rendered using PyMol. c) The IC_50_ values for PF04217903 and PF04217903-TCO (**15**) against recombinant MET were determined using the z′-lyte kinase assay. Data were fit to a sigmoidal dose-response curve using GraphPad software (Prism).
(TIFF)

Figure S3 **Comparison of two-step (top row) and one-step (bottom row) labeling.** OVCA429 cells were incubated for 30 min with Foretinib-TCO (**11**) or Foretinib-BODIPY-FL (**12**). Cells were washed and then incubated for 30 min with 1 μM CFDA-Tz for bioorthogonal reaction inside living cells (a-d only). 40x images were collected using a DeltaVision microscope. a–c) Cells were treated with 1000, 200, and 40 nM, respectively, of Foretinib-TCO (**11**)/Tz-CFDA; d–f) Cells were treated with 1000, 200 and 40 nM, respectively, of Foretinib-BODIPY-FL (**12**). Scale bar: 10 μm.
(TIFF)

Figure S4 **Western blot analysis of MET, PDGFRα, AXL, RON and KDR expression in 8 different cell lines including, A2780 (**1**), OVCA429 (**2**), SK-BR-3 (**3**), MDA-MB-436 (**4**), MDA-MB-231 (**5**), HCC1937 (**6**), HCC1395 (**7**) and HCC38 (**8**).**
(TIFF)

Figure S5 **MET imaging in OVCA429 cells.** Cells were incubated for 30 min with 40 nM Foretinib (a,b and c), PF04217903-TCO (**15**) (d, e and f) or Foretinib-TCO (**11**) (g, h and i), washed, and incubated for 30 min with 1 μM Tz-CFDA for bioorthogonal reaction inside living cells. After fixation with 2% paraformaldehyde, MET was labeled using a MET primary antibody and AlexaFluor 647 labeled secondary antibody (i-l). After nuclear staining with Hoechst 33342 (blue nuclei) for 10 min, 40X images were collected using a DeltaVision microscope. Note the striking co-localization between the selective MET imaging agent and the MET antibody stain. Foretinib-TCO shows a much broader intracellular distribution. Scale bar: 10 μm.
(TIFF)

Figure S6 **Live cell fluorescence microscopic imaging of Foretinib-TCO (11)/Tz-CFDA (**a**, **b**) or PF04217903-TCO (**15**)/Tz-CFDA (**c**, **d**) in OVCA429 cells.** Cells were incubated for 30 min with 1 μM Foretinib-TCO (**11**) or 40 nM PF04217903-TCO (**15**). Cells were then washed and incubated for 30 min with 1 μM Tz-CFDA for bioorthogonal reaction inside living cells. After washing, live cells were imaged in a humidified environmental chamber of a DeltaVision microscope using a 40X objective. Scale bar: 10 μm.
(TIFF)

File S1 **NMR-spectra of all the products.**
(PDF)
