# Supplementary figures and images for: Bioorthogonal Small Molecule Imaging Agents Allow Single-Cell Imaging of MET
Source: PLoS One. 2013 Nov 12;8(11):e81275. doi: 10.1371/journal.pone.0081275 (PMC3827223; doi:10.1371/journal.pone.0081275)

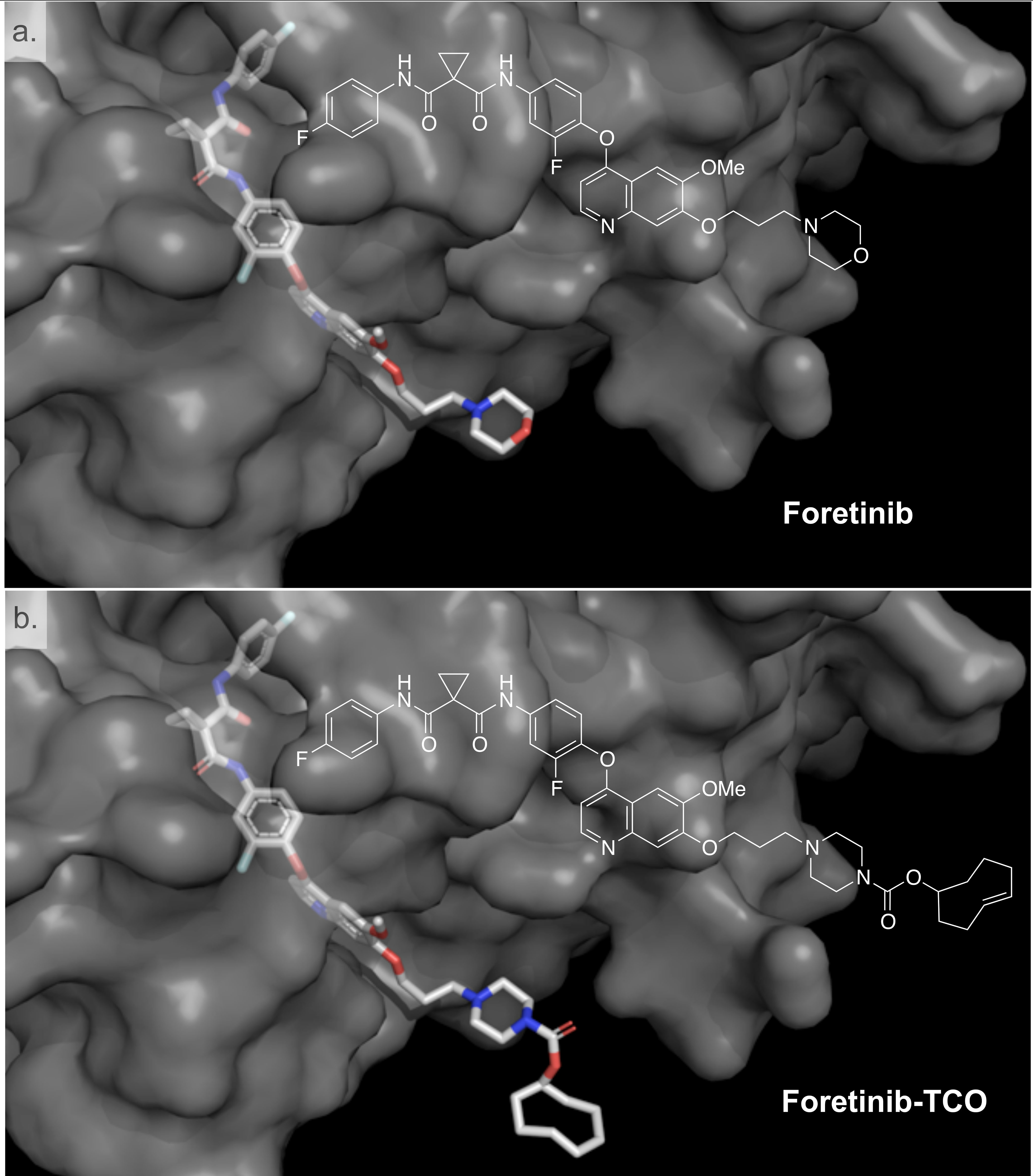

Supplement: Figure S1 — Molecular design of polypharmacology companion imaging drugs (PCID) based on the crystal structures of Foretinib. a) Crystal structure of MET in complex with Foretinib (PDB ID: 3LQ8). b) Crystal structure prediction of MET in complex with Foretinib-TCO. 3D models were rendered using PyMol. (TIFF) [file pone.0081275.s001.tiff]

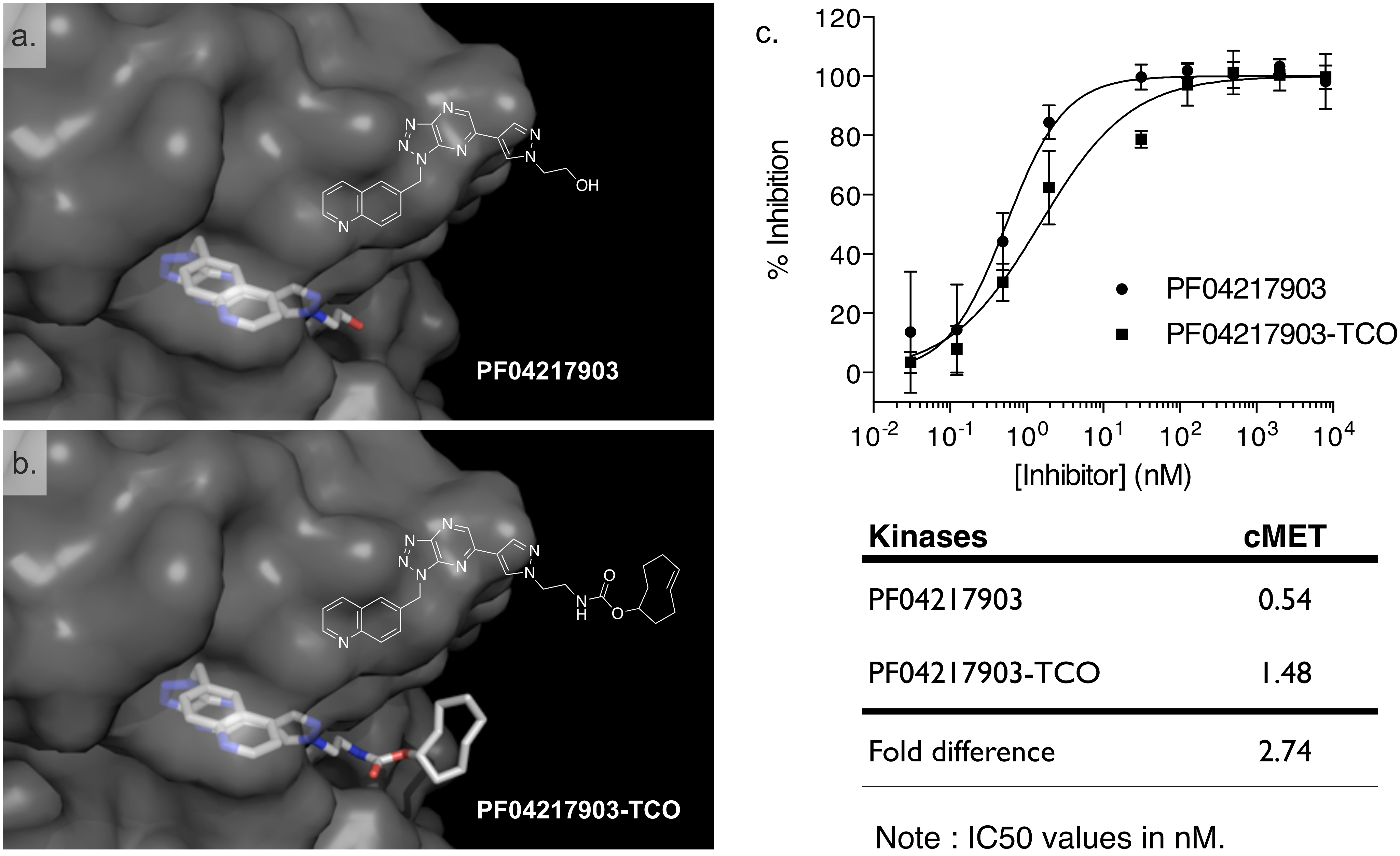

Supplement: Figure S2 — Molecular analysis of MET-specific imaging drugs based on the PF04217903 scaffold. a) Crystal structure of MET in complex with PF04217903 (PDB ID: 3zxz). b) Crystal structure prediction of MET in complex with PF04217903-TCO. 3D models were rendered using PyMol. c) The IC50 values for PF04217903 and PF04217903-TCO (15) against recombinant MET were determined using the z′-lyte kinase assay. Data were fit to a sigmoidal dose-response curve using GraphPad software (Prism). (TIFF) [file pone.0081275.s002.tiff]

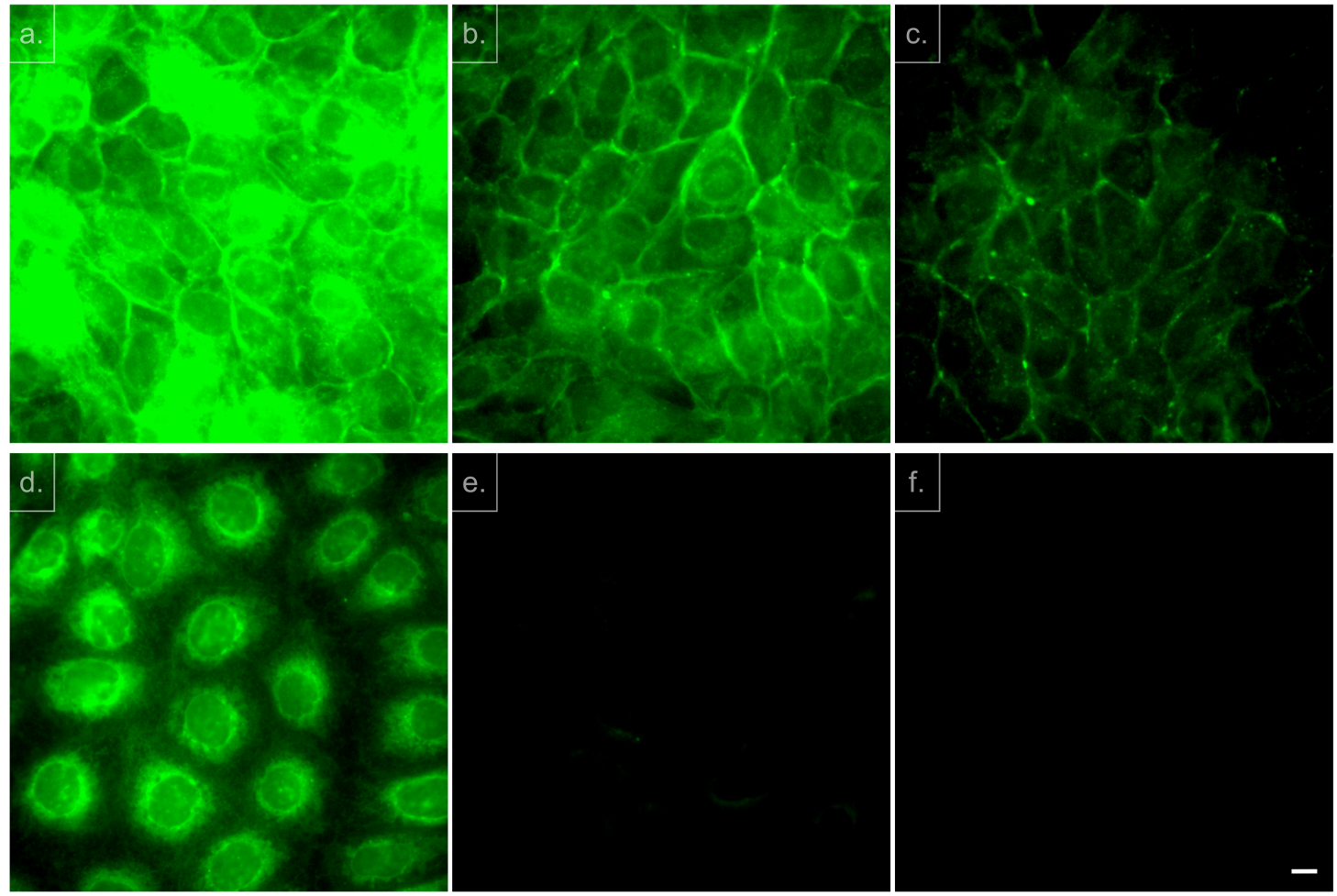

Supplement: Figure S3 — Comparison of two-step (top row) and one-step (bottom row) labeling. OVCA429 cells were incubated for 30 min with Foretinib-TCO (11) or Foretinib-BODIPY-FL (12). Cells were washed and then incubated for 30 min with 1 μM CFDA-Tz for bioorthogonal reaction inside living cells (a-d only). 40x images were collected using a DeltaVision microscope. a–c) Cells were treated with 1000, 200, and 40 nM, respectively, of Foretinib-TCO (11)/Tz-CFDA; d–f) Cells were treated with 1000, 200 and 40 nM, respectively, of Foretinib-BODIPY-FL (12). Scale bar: 10 μm. (TIFF) [file pone.0081275.s003.tiff]

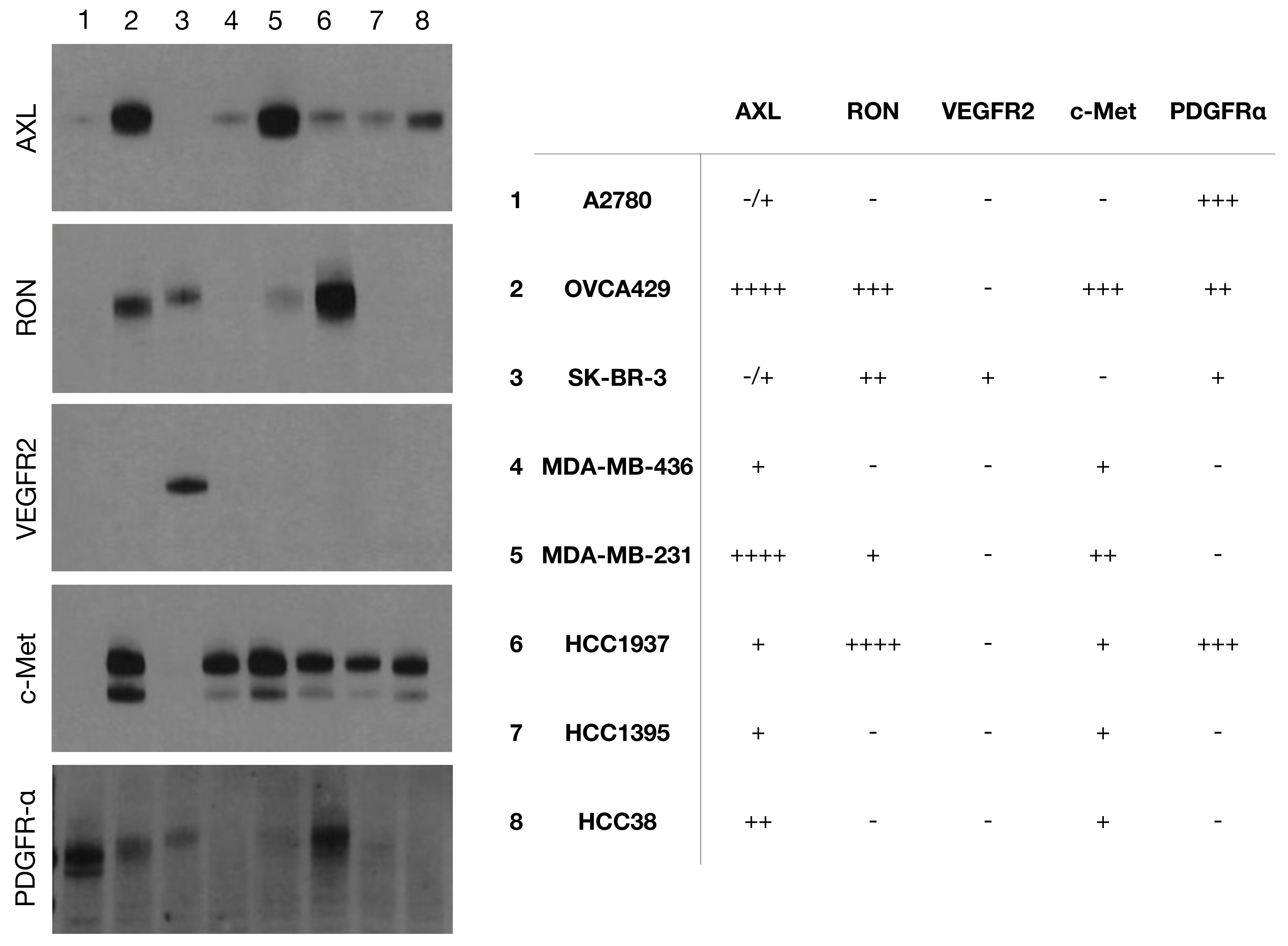

Supplement: Figure S4 — Western blot analysis of MET, PDGFRα, AXL, RON and KDR expression in 8 different cell lines including, A2780 (1), OVCA429 (2), SK-BR-3 (3), MDA-MB-436 (4), MDA-MB-231 (5), HCC1937 (6), HCC1395 (7) and HCC38 (8). (TIFF) [file pone.0081275.s004.tiff]

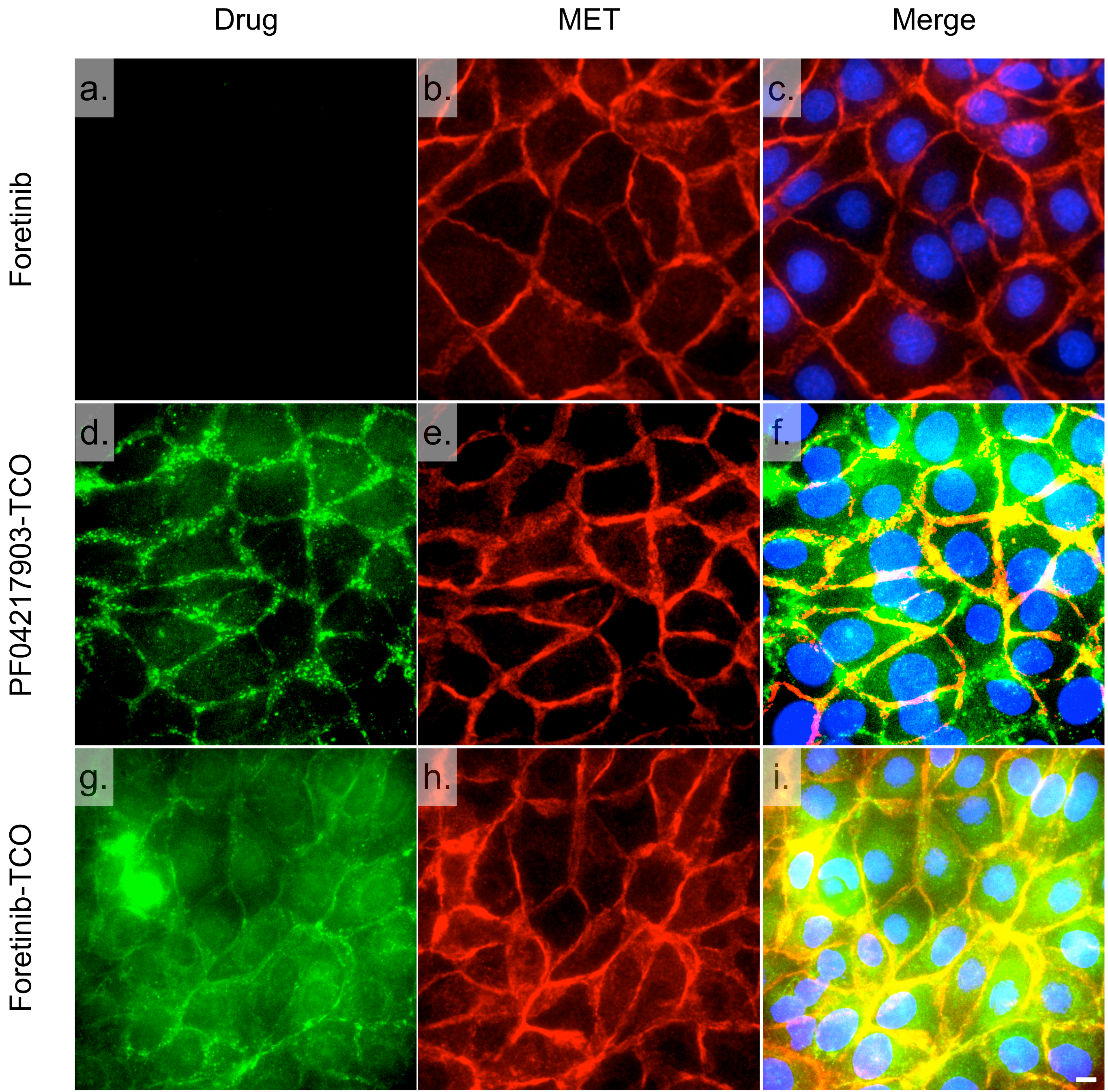

Supplement: Figure S5 — MET imaging in OVCA429 cells. Cells were incubated for 30 min with 40 nM Foretinib (a,b and c), PF04217903-TCO (15) (d, e and f) or Foretinib-TCO (11) (g, h and i), washed, and incubated for 30 min with 1 μM Tz-CFDA for bioorthogonal reaction inside living cells. After fixation with 2% paraformaldehyde, MET was labeled using a MET primary antibody and AlexaFluor 647 labeled secondary antibody (i-l). After nuclear staining with Hoechst 33342 (blue nuclei) for 10 min, 40X images were collected using a DeltaVision microscope. Note the striking co-localization between the selective MET imaging agent and the MET antibody stain. Foretinib-TCO shows a much broader intracellular distribution. Scale bar: 10 μm. (TIFF) [file pone.0081275.s005.tiff]

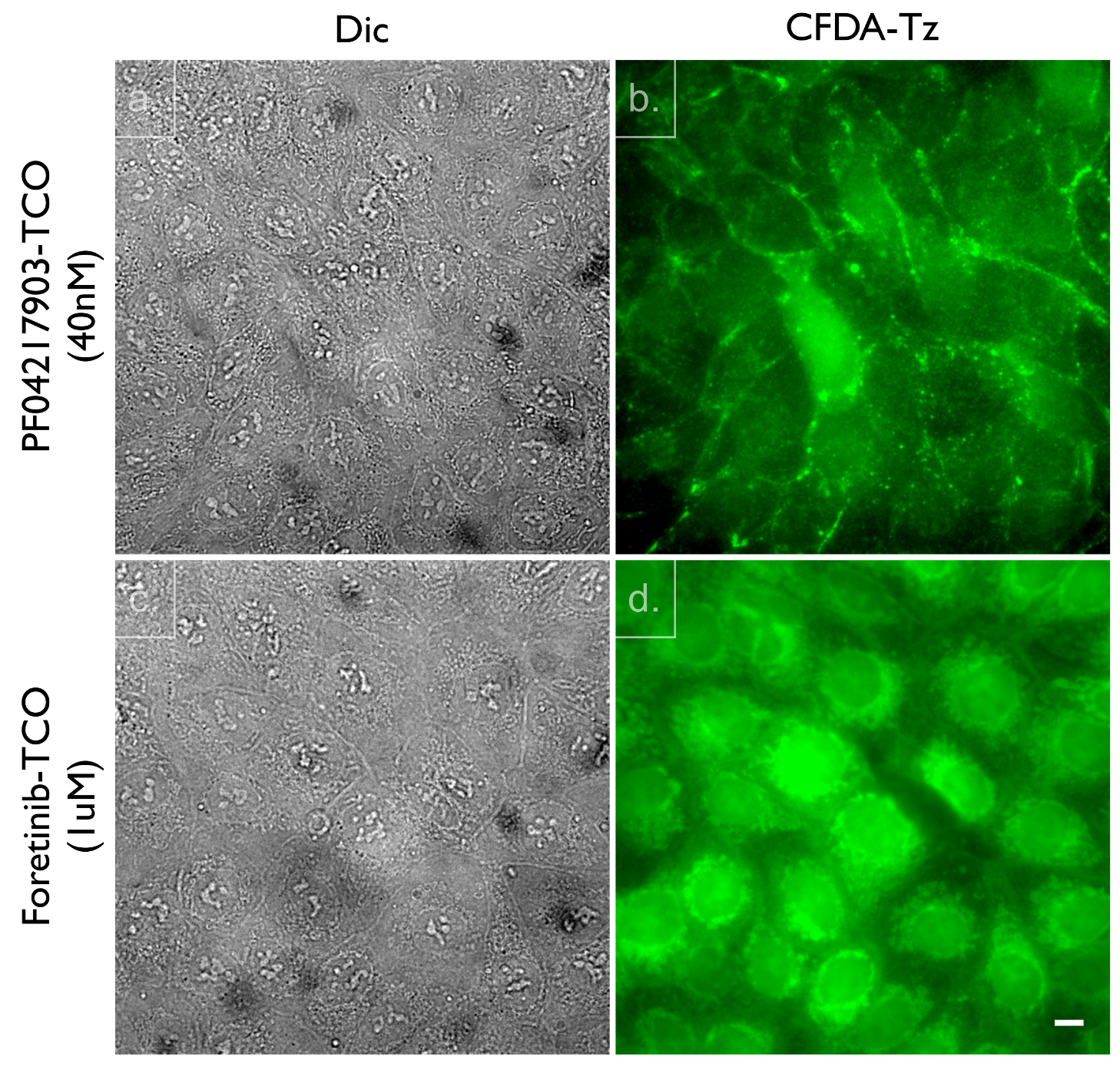

Supplement: Figure S6 — Live cell fluorescence microscopic imaging of Foretinib-TCO (11)/Tz-CFDA (a, b) or PF04217903-TCO (15)/Tz-CFDA (c, d) in OVCA429 cells. Cells were incubated for 30 min with 1 μM Foretinib-TCO (11) or 40 nM PF04217903-TCO (15). Cells were then washed and incubated for 30 min with 1 μM Tz-CFDA for bioorthogonal reaction inside living cells. After washing, live cells were imaged in a humidified environmental chamber of a DeltaVision microscope using a 40X objective. Scale bar: 10 μm. (TIFF) [file pone.0081275.s006.tiff]
