# Supplementary material for: Bioorthogonal Small Molecule Imaging Agents Allow Single-Cell Imaging of MET
Source: PLoS One. 2013 Nov 12;8(11):e81275. doi: 10.1371/journal.pone.0081275 (PMC3827223; doi:10.1371/journal.pone.0081275)

Spectra ^{1}H and ^{13}C of compound 1

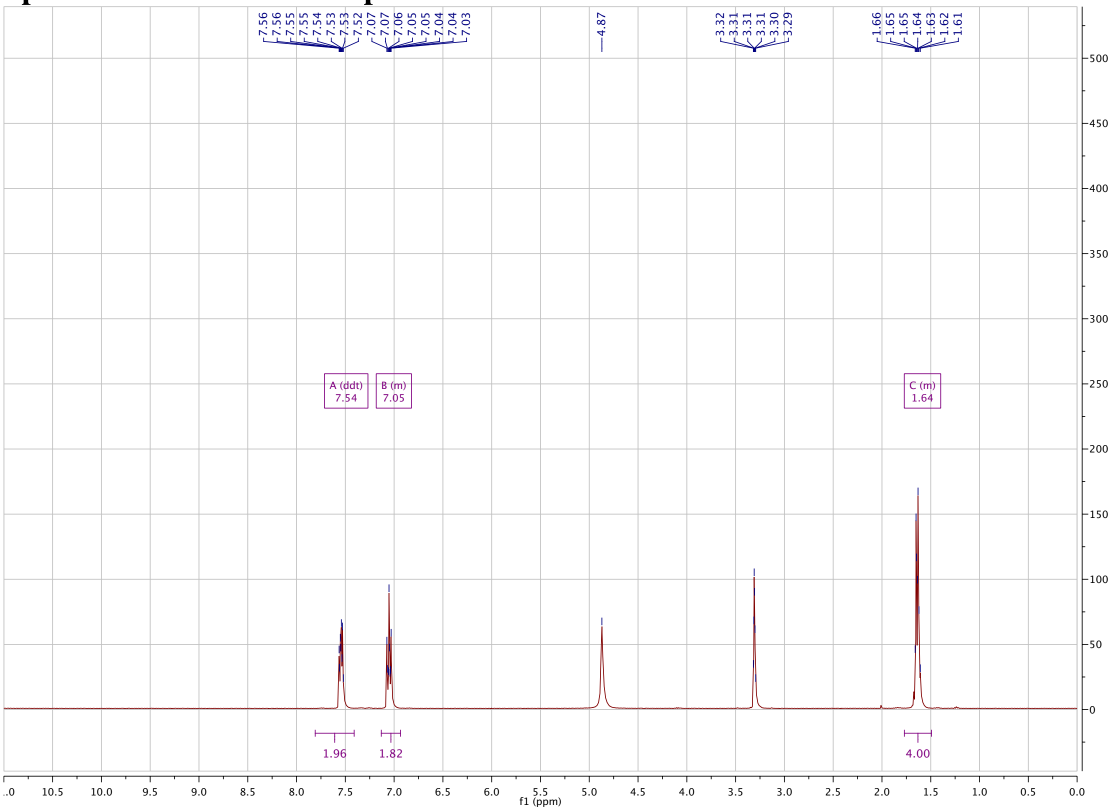

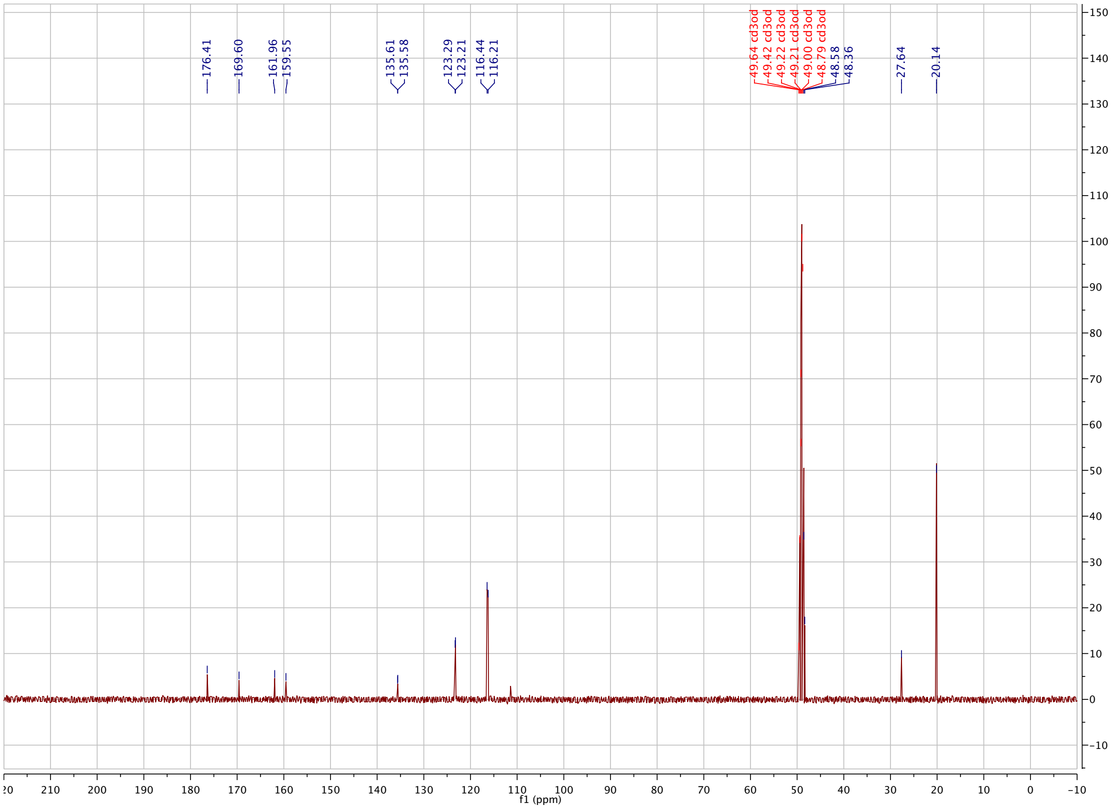

Spectra ¹H and ¹³C of compound 2

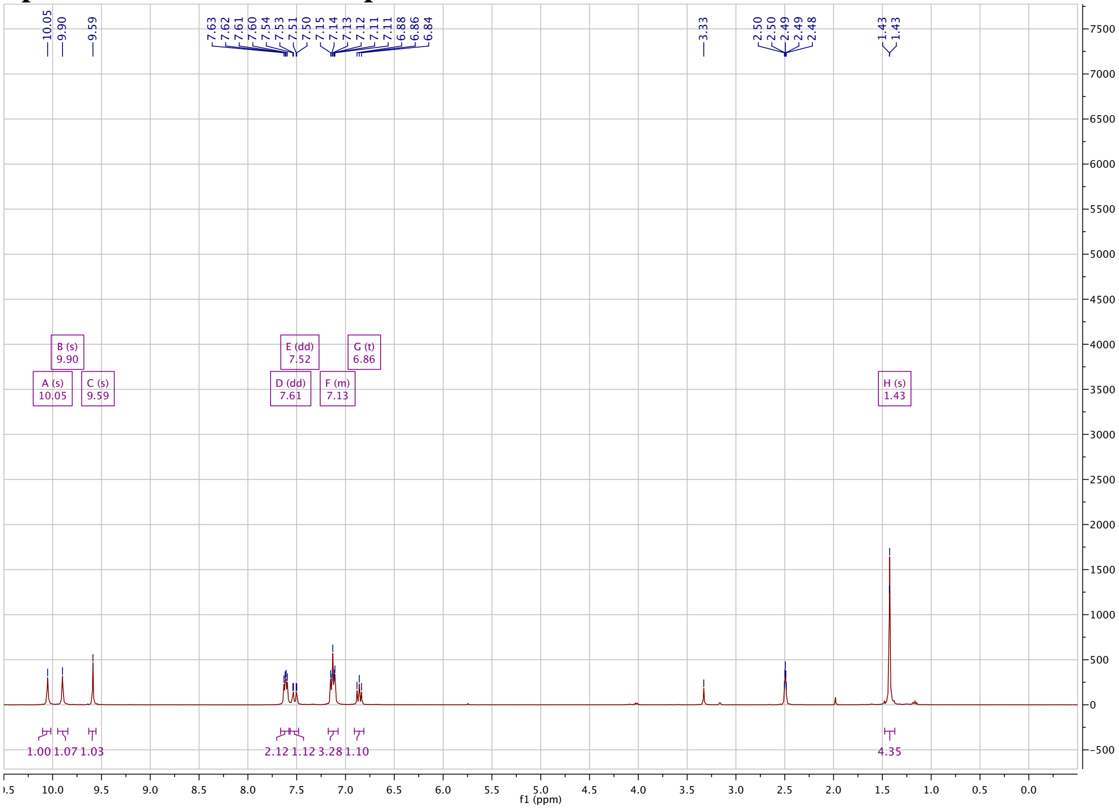

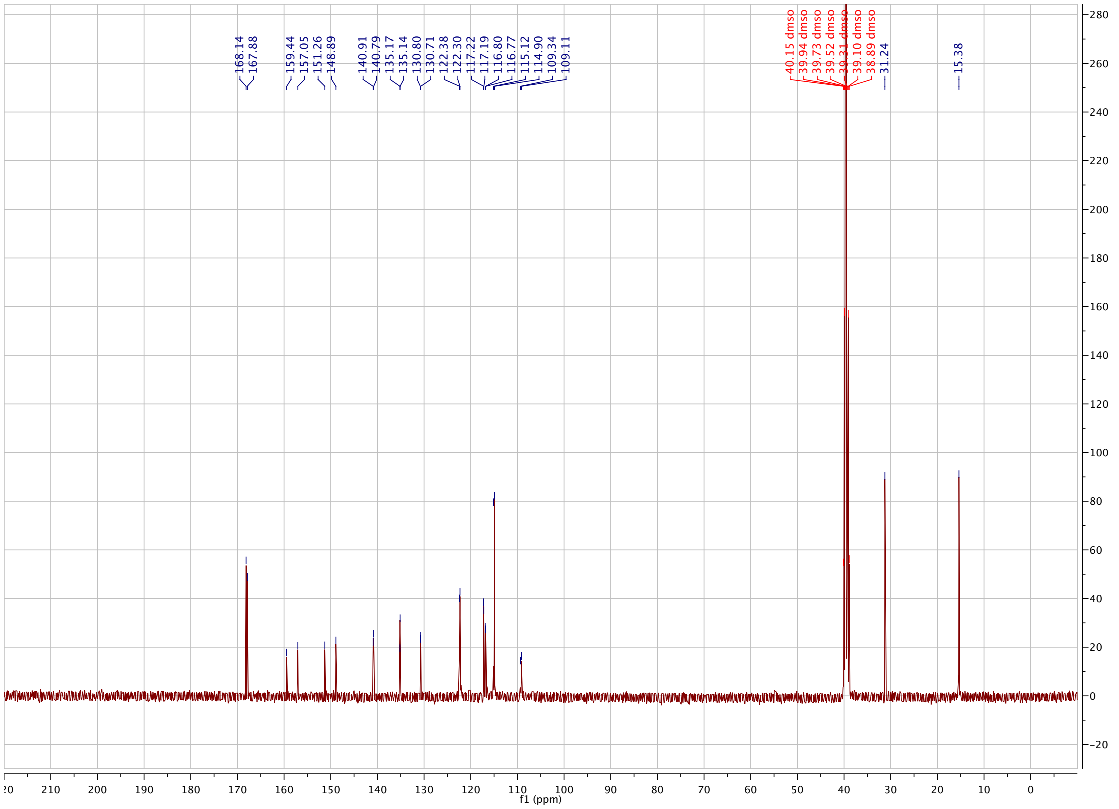

Spectra ^{1}H and ^{13}C of compound 3

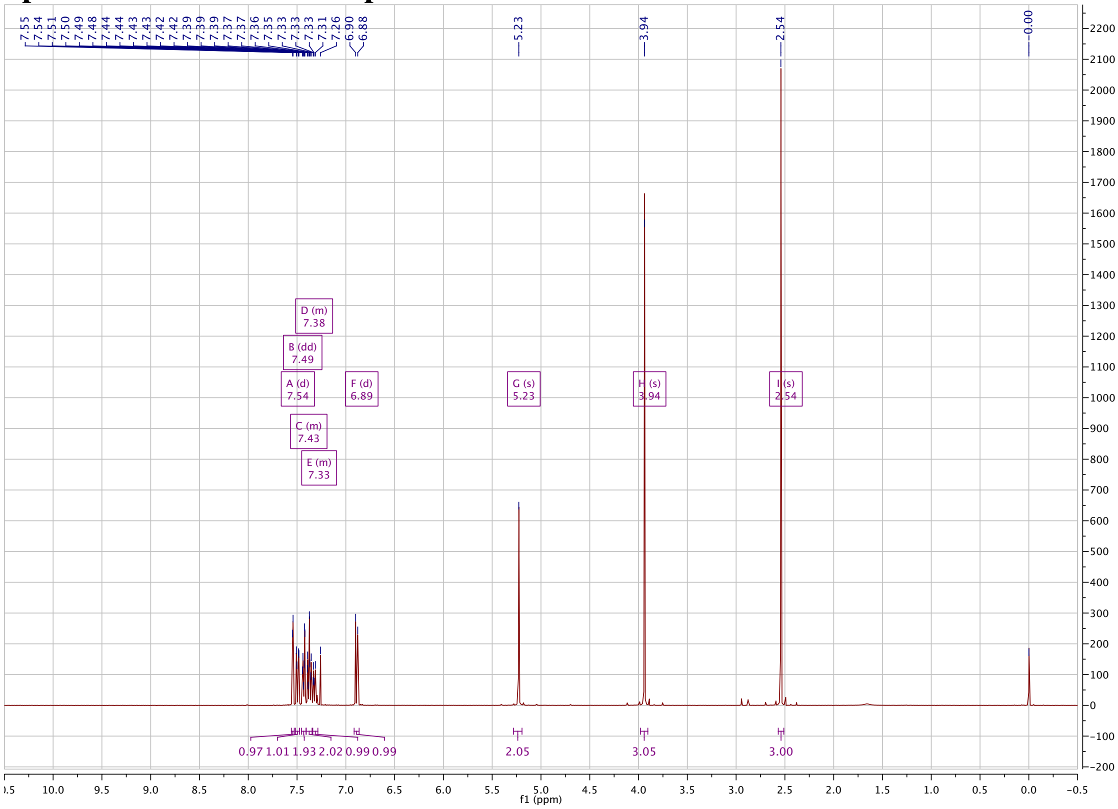

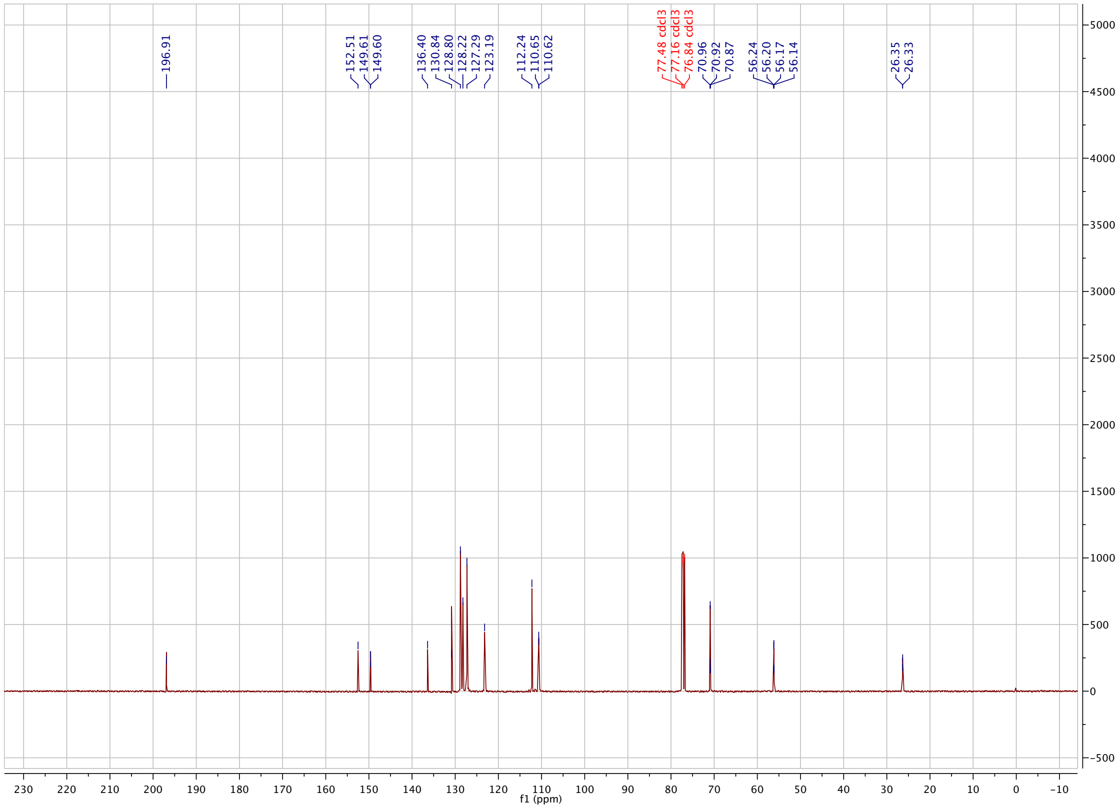

Spectra ^{1}H and ^{13}C of compound 4

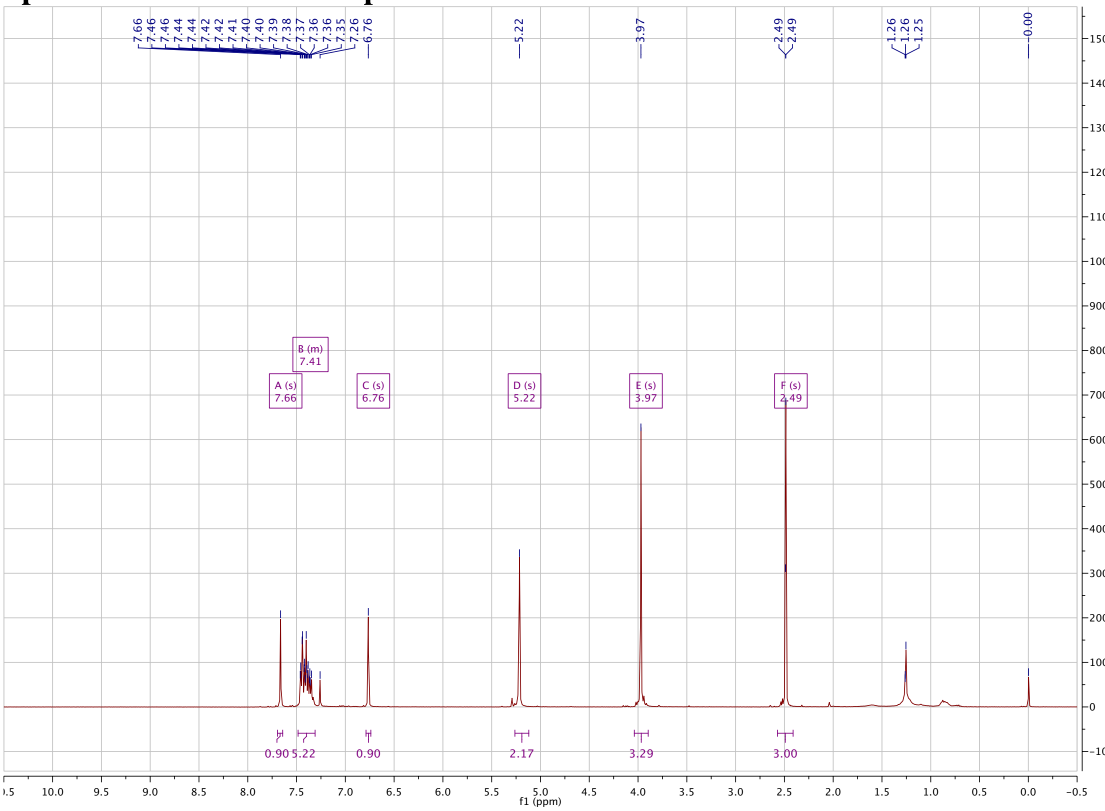

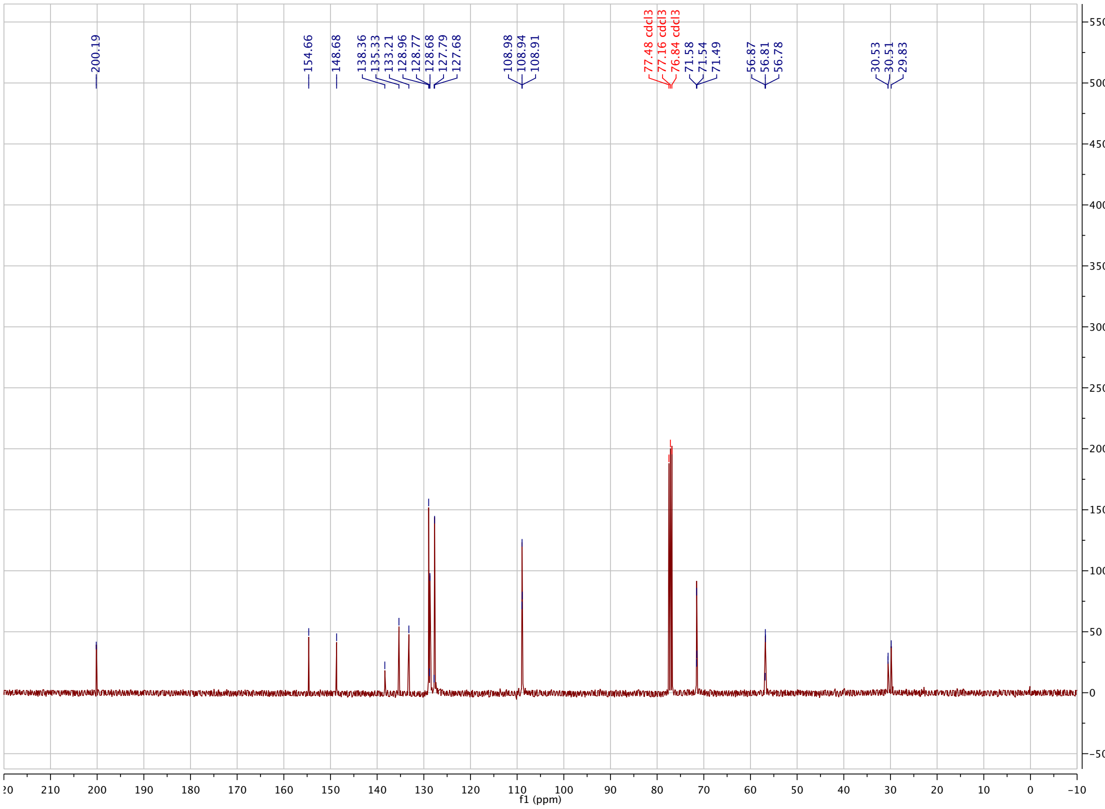

Spectra ^{1}H and ^{13}C of compound 5

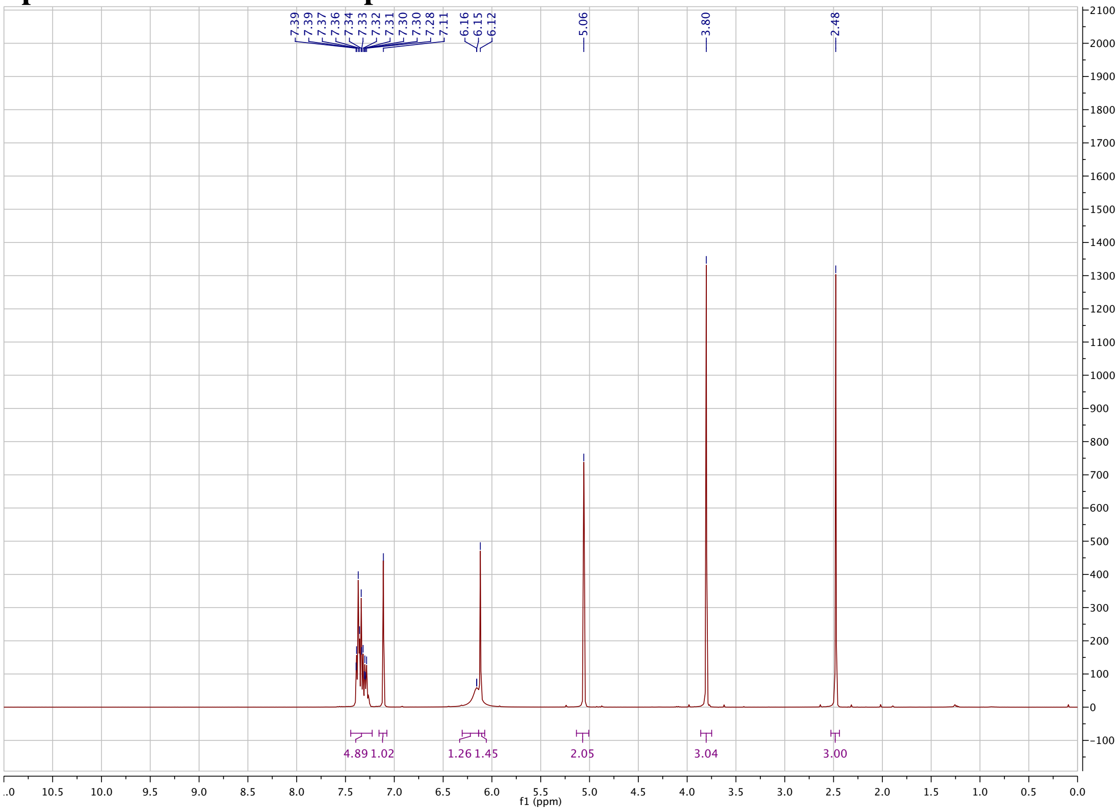

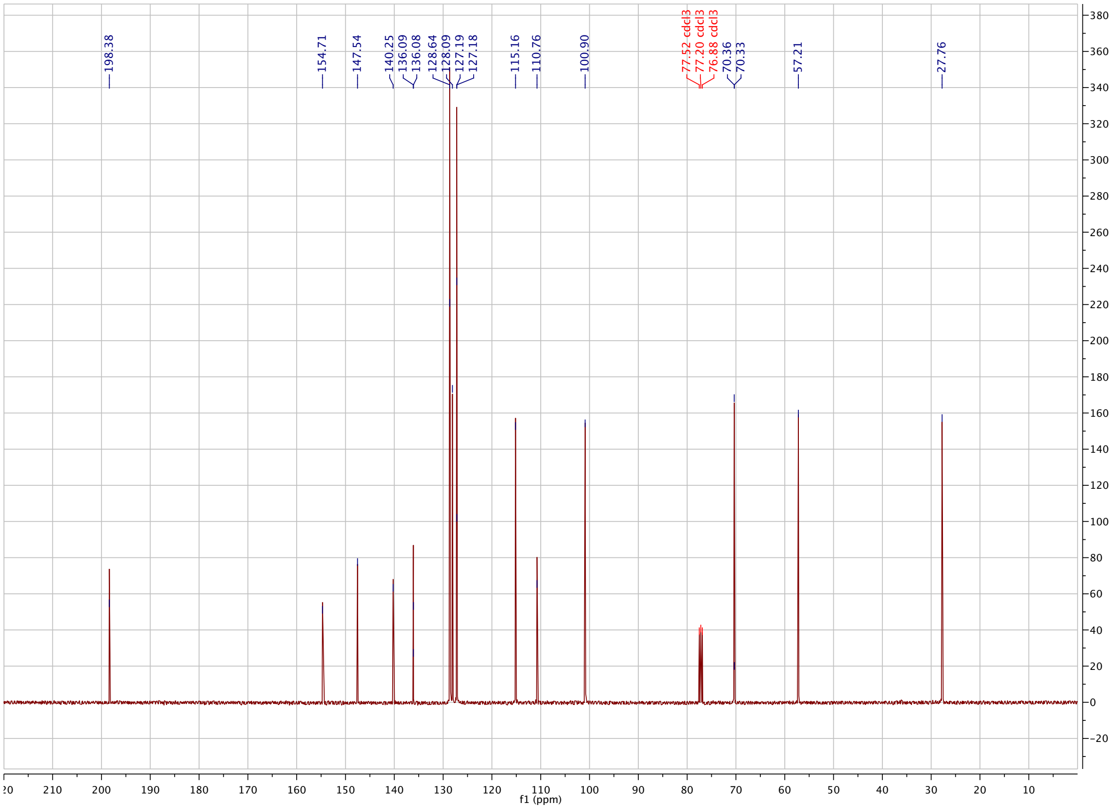

Spectra ¹H and ¹³C of compound 6

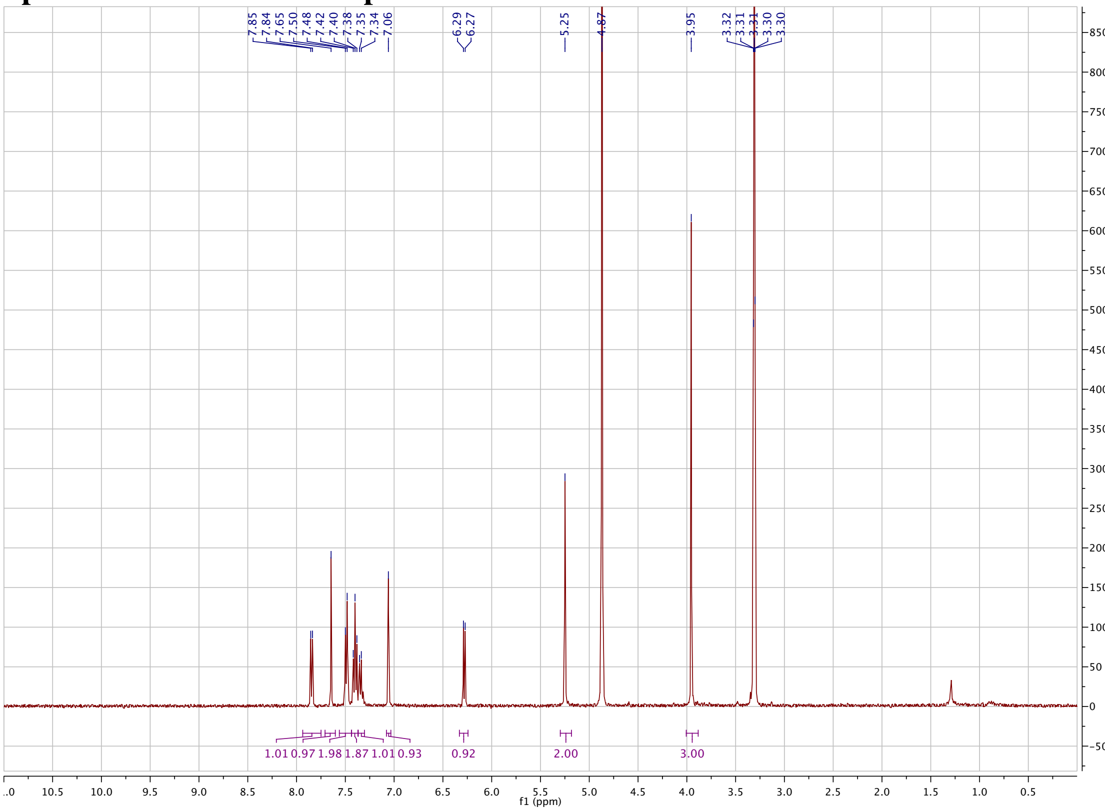

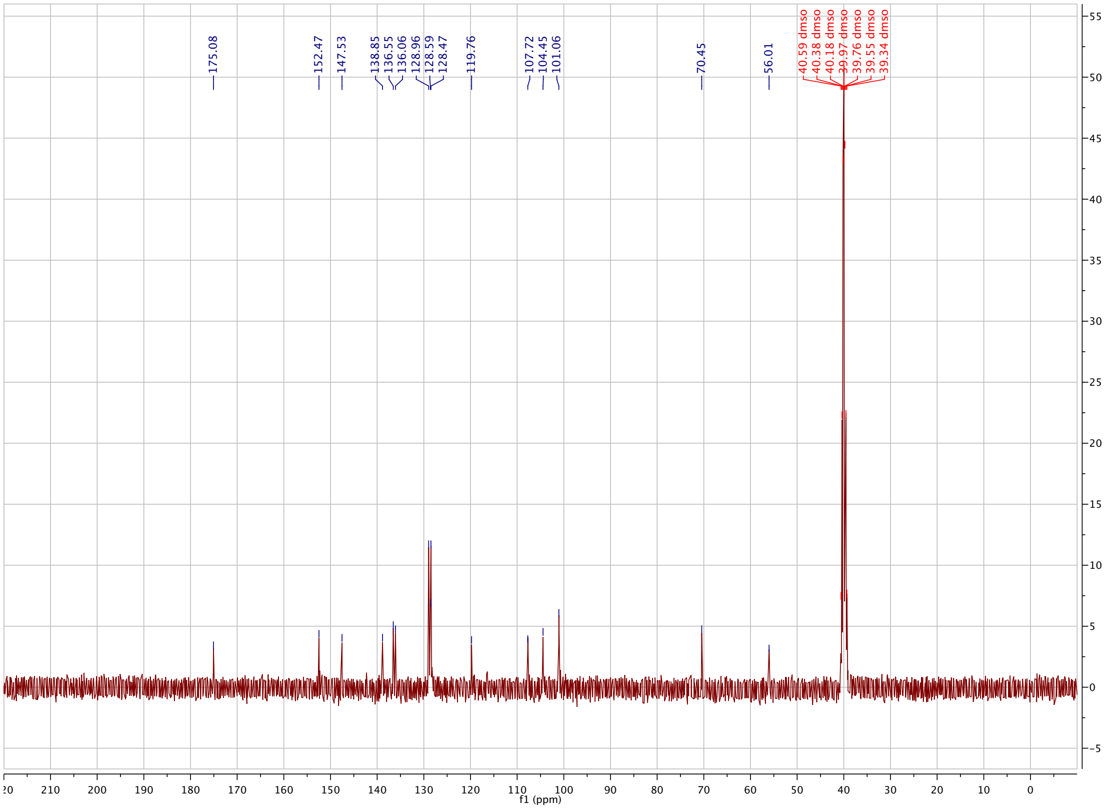

Spectra ¹H of compound 8

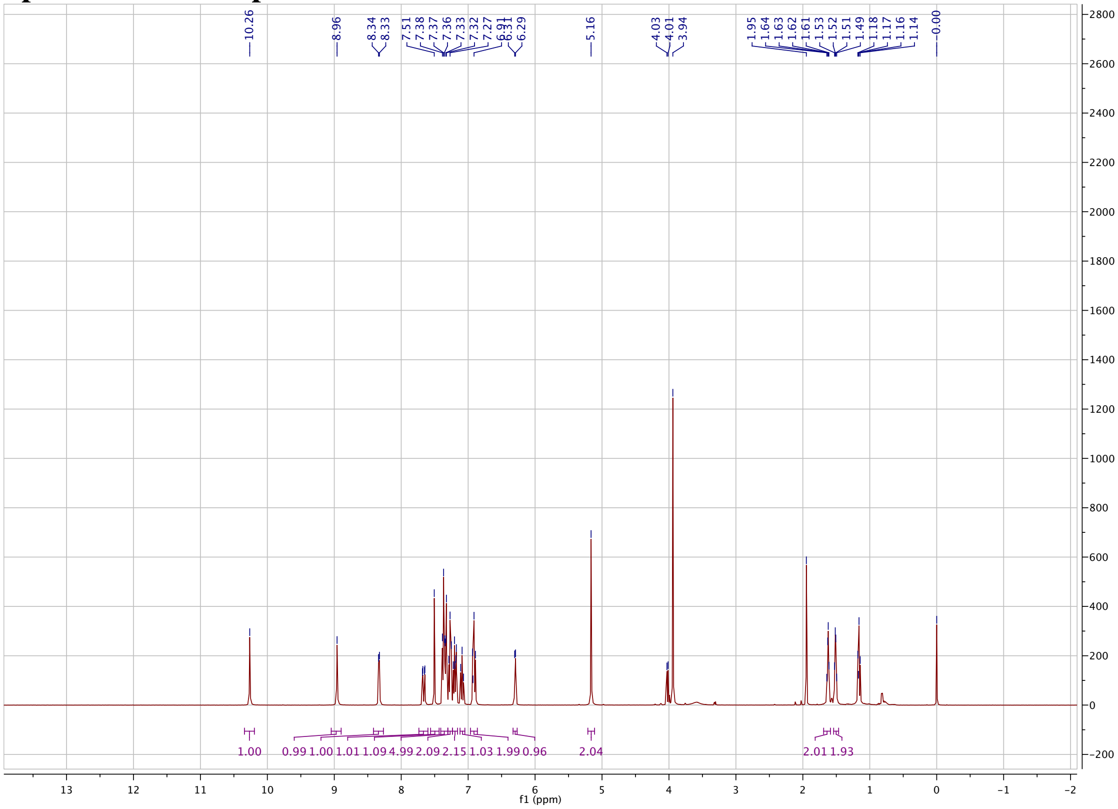

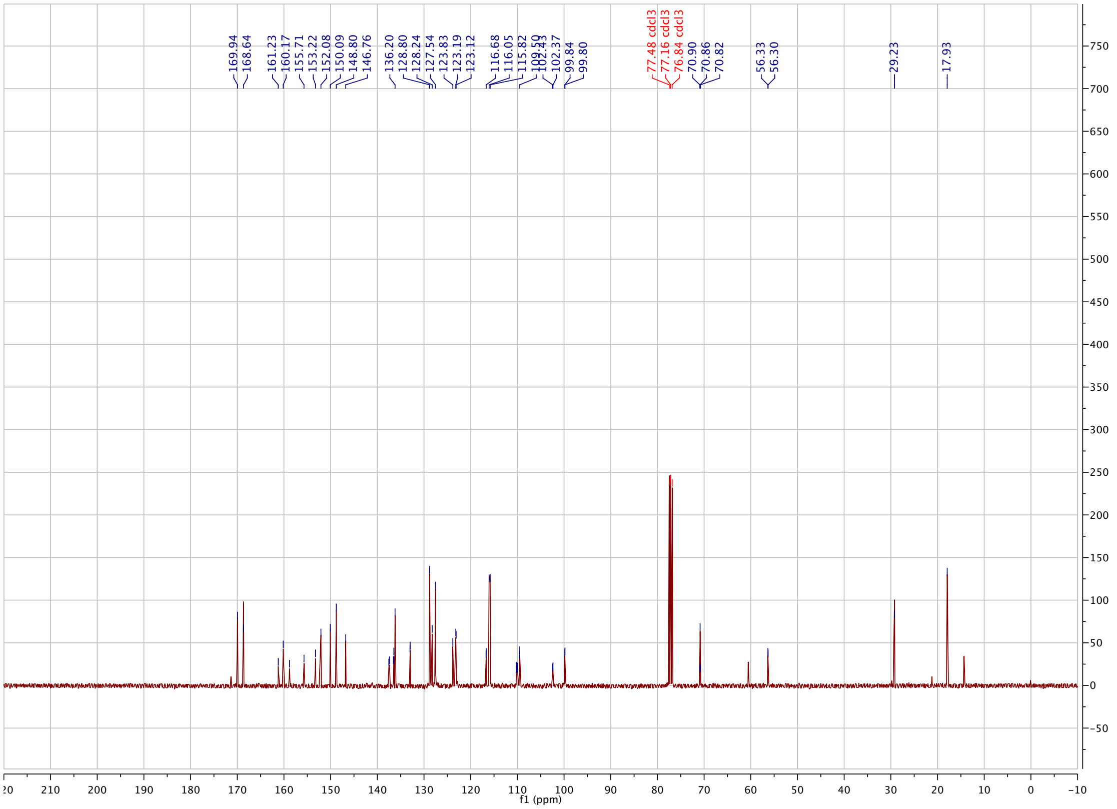

Spectra ^{1}H and ^{13}C of compound tert-butyl 4-(3-bromopropyl)piperazine-1-carboxylate

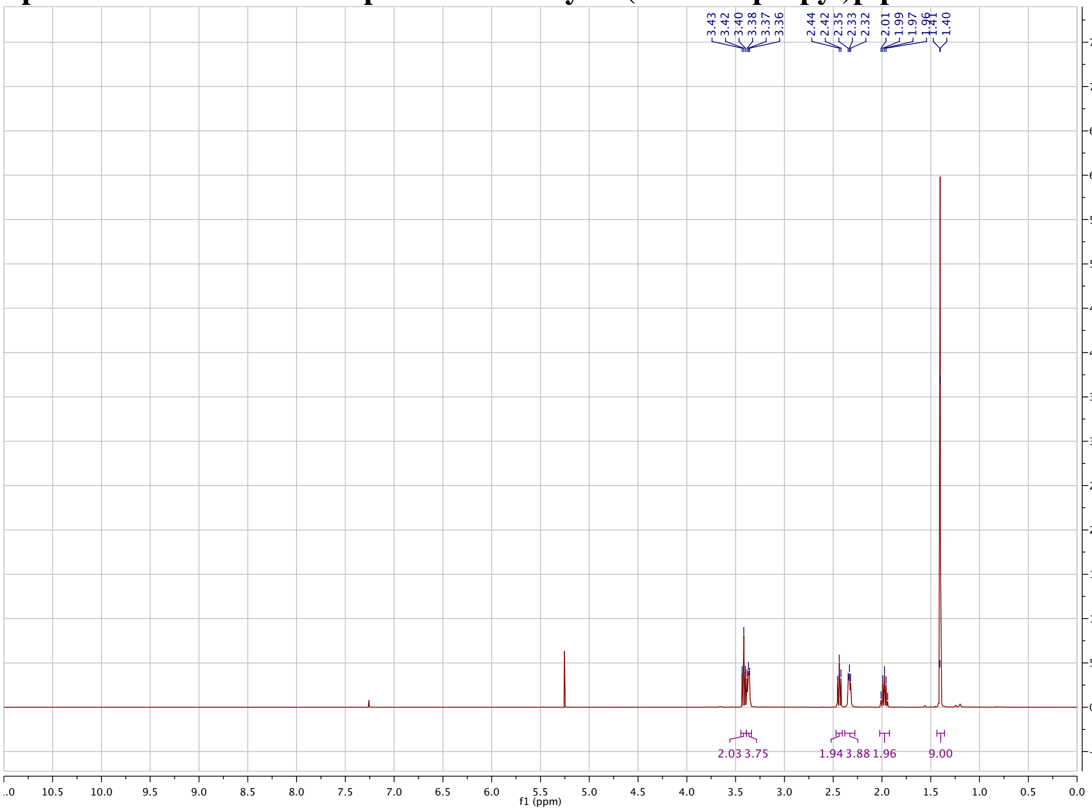

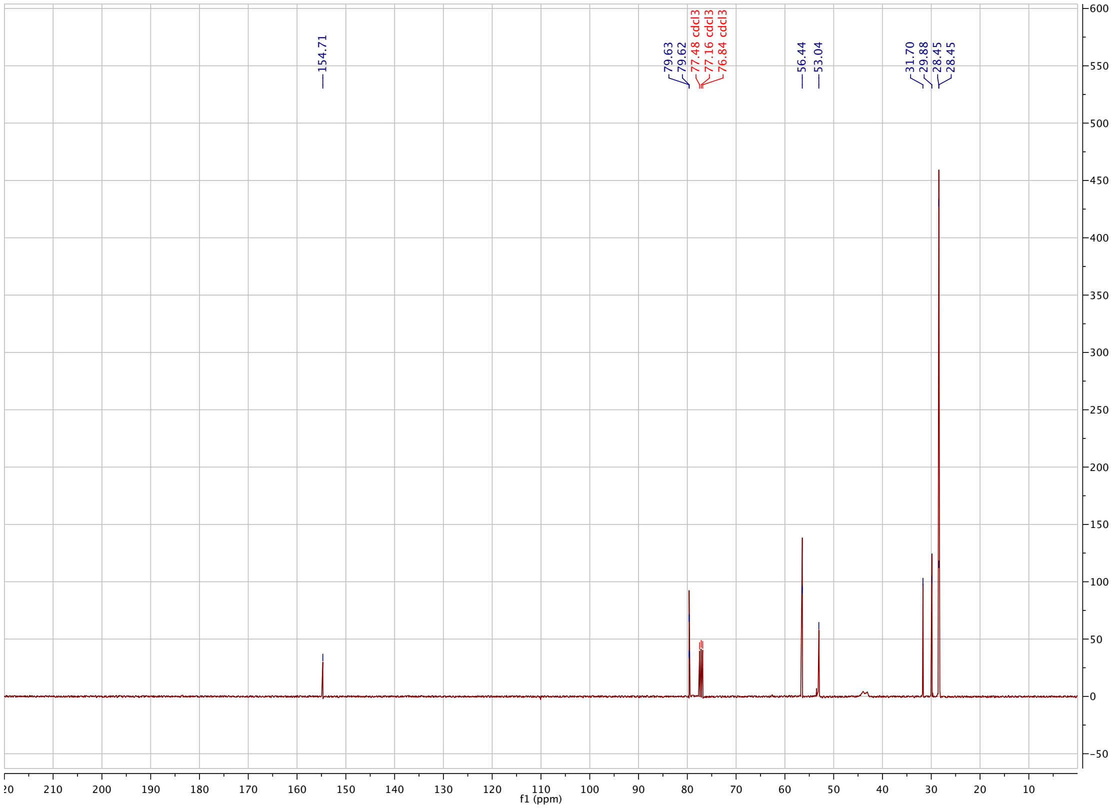

Spectra ¹H and ¹³C of compound 9

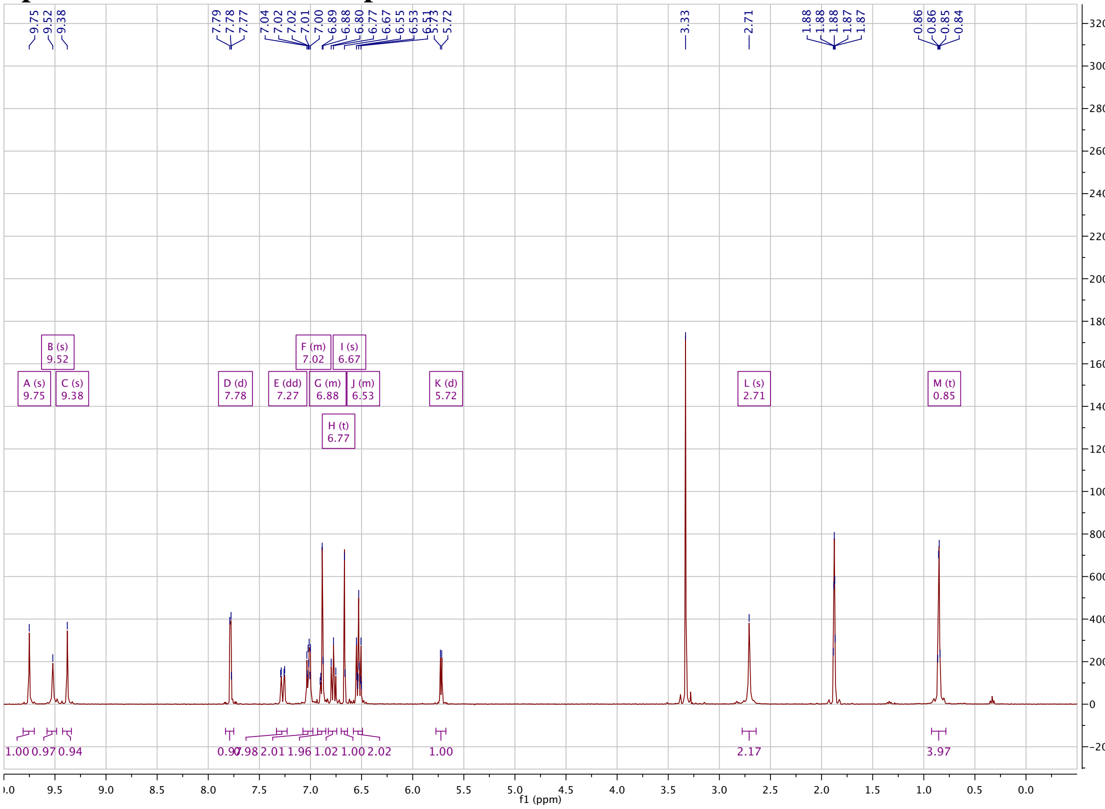

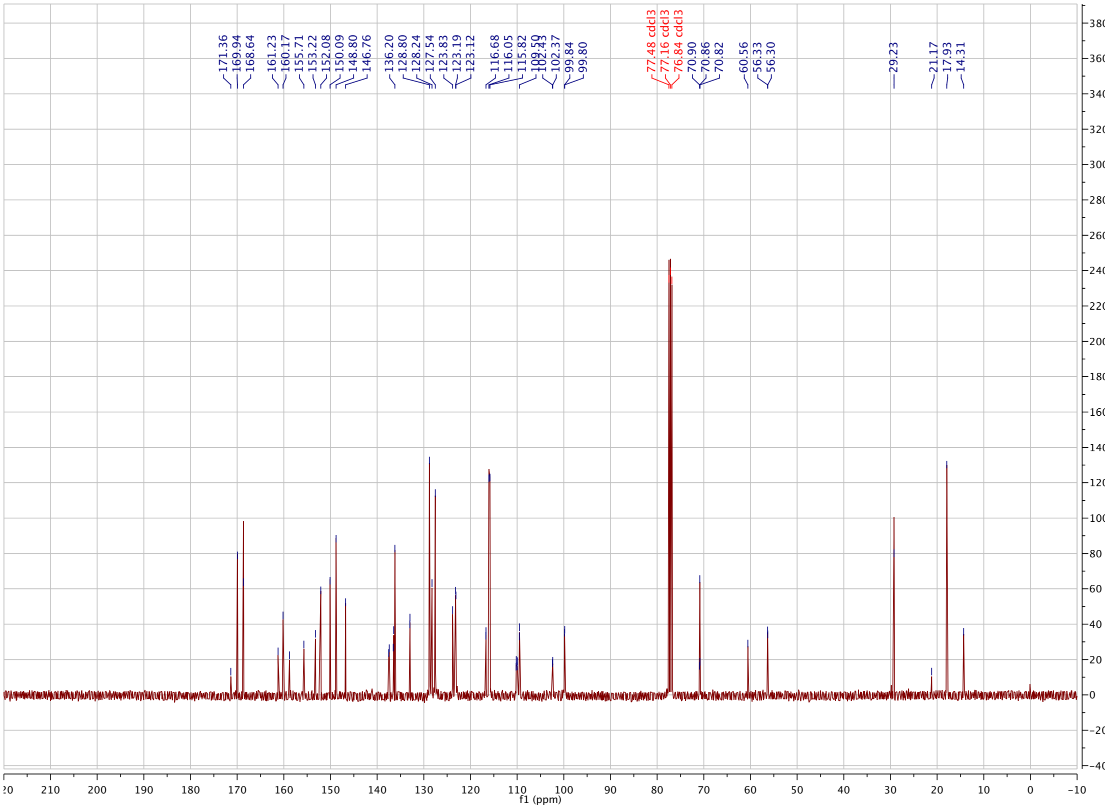

Spectra ^{1}H of compound 10

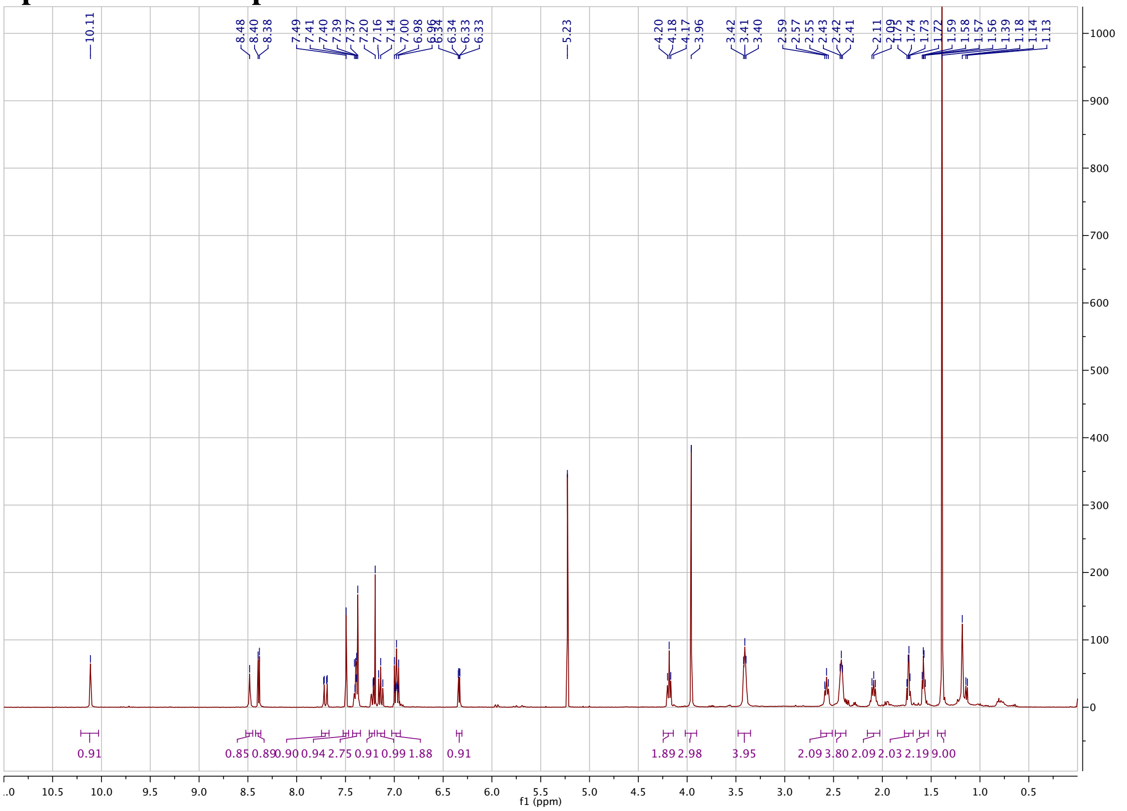

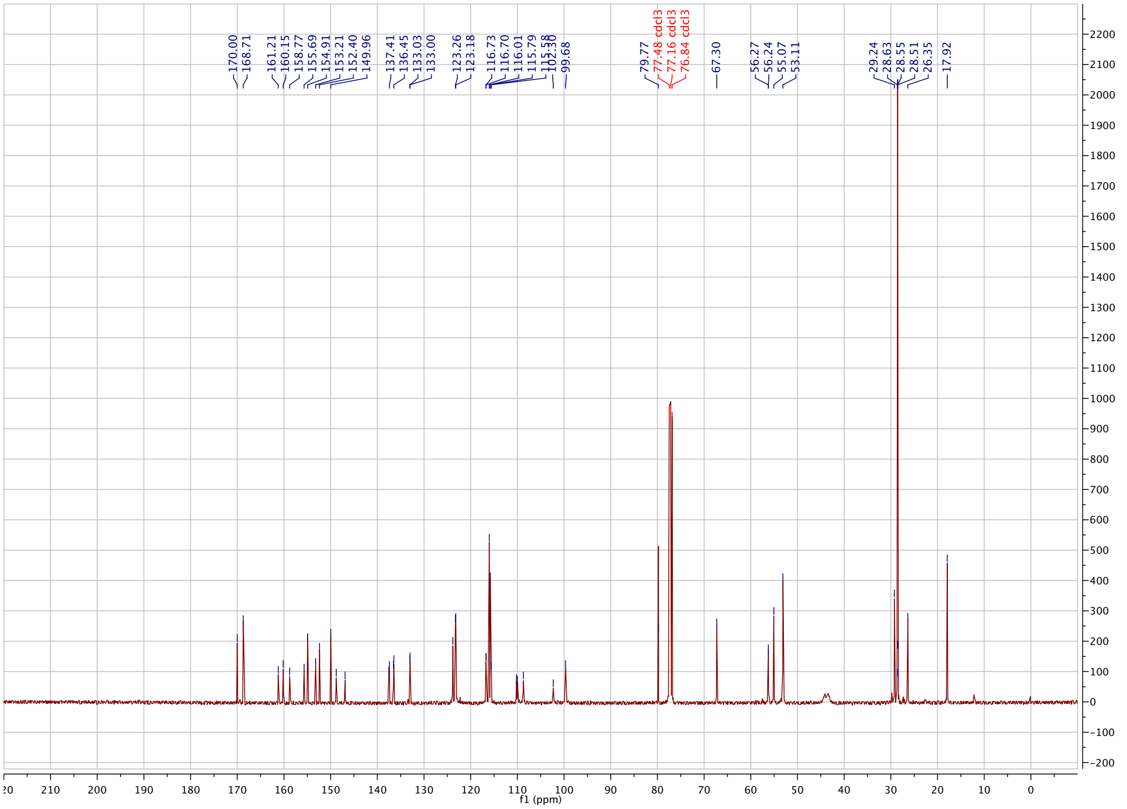

Spectra ^{1}H and ^{13}C of compound 11

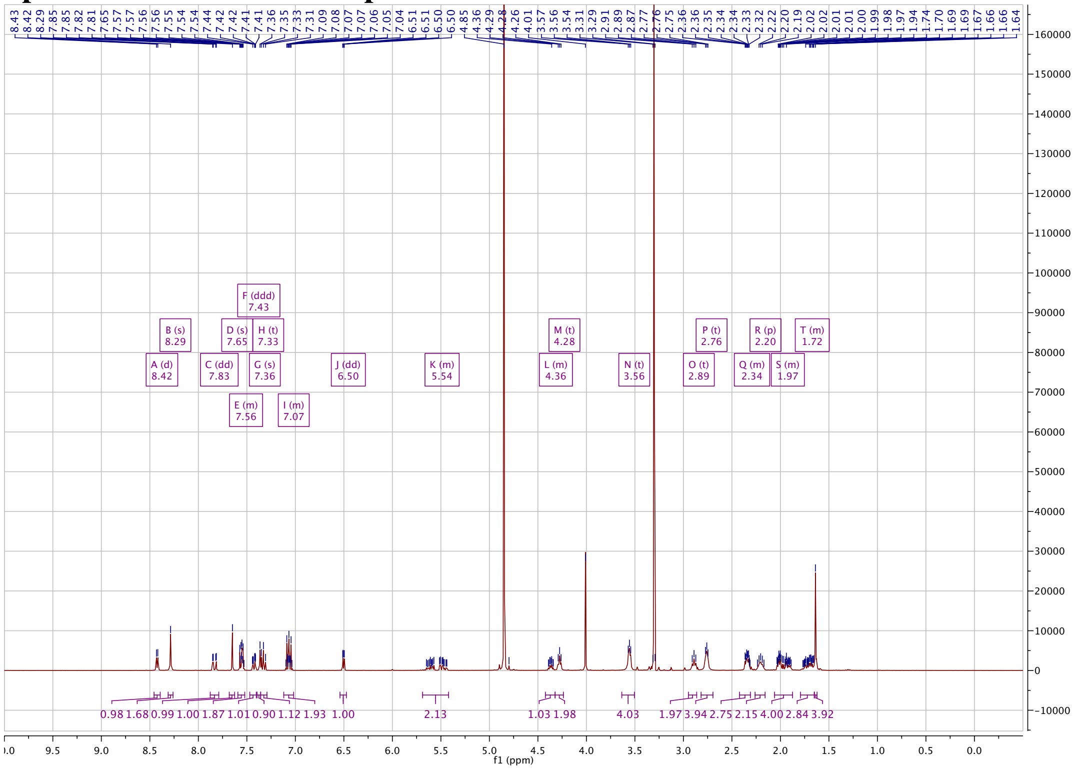

Spectra ^{1}H and ^{13}C of compound 12

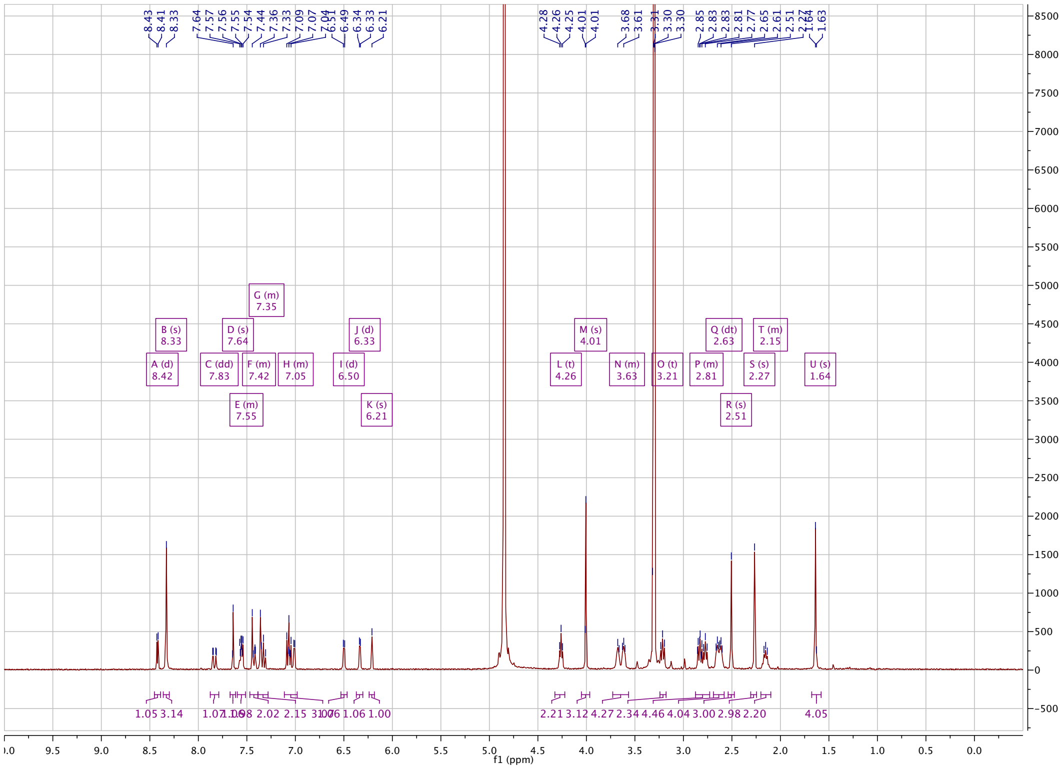

Spectra ¹H and ¹³C of compound 13

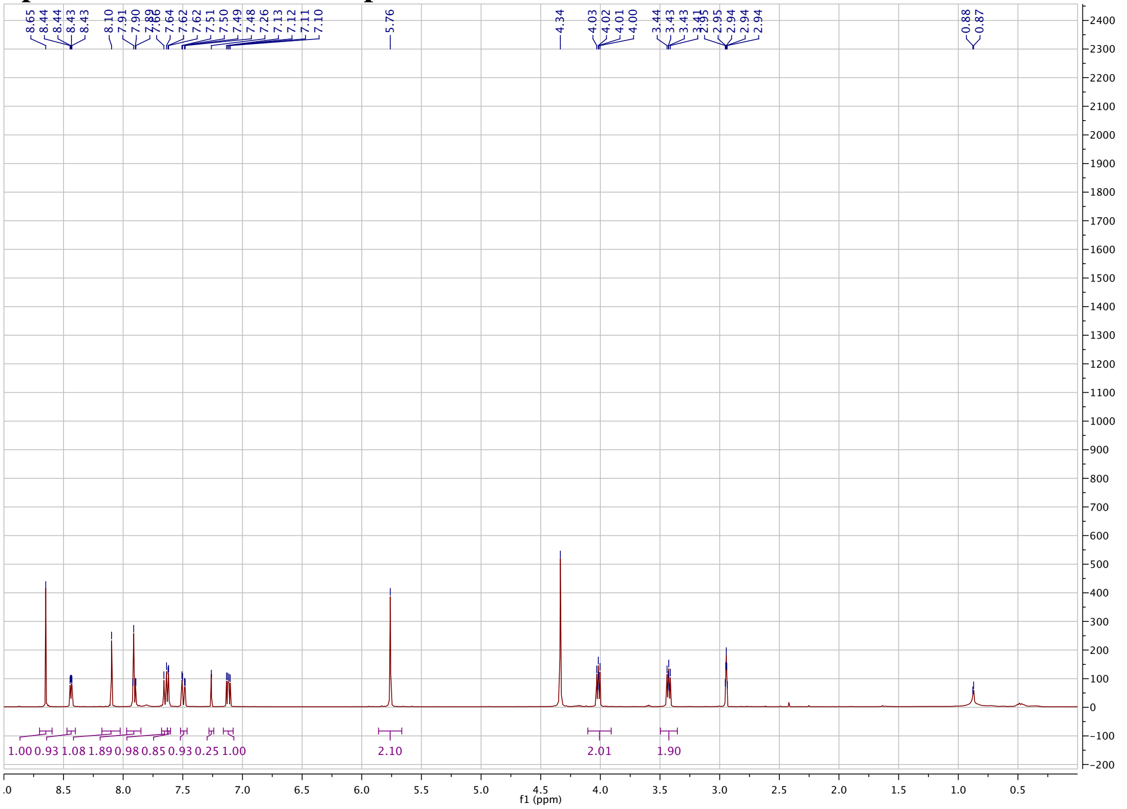

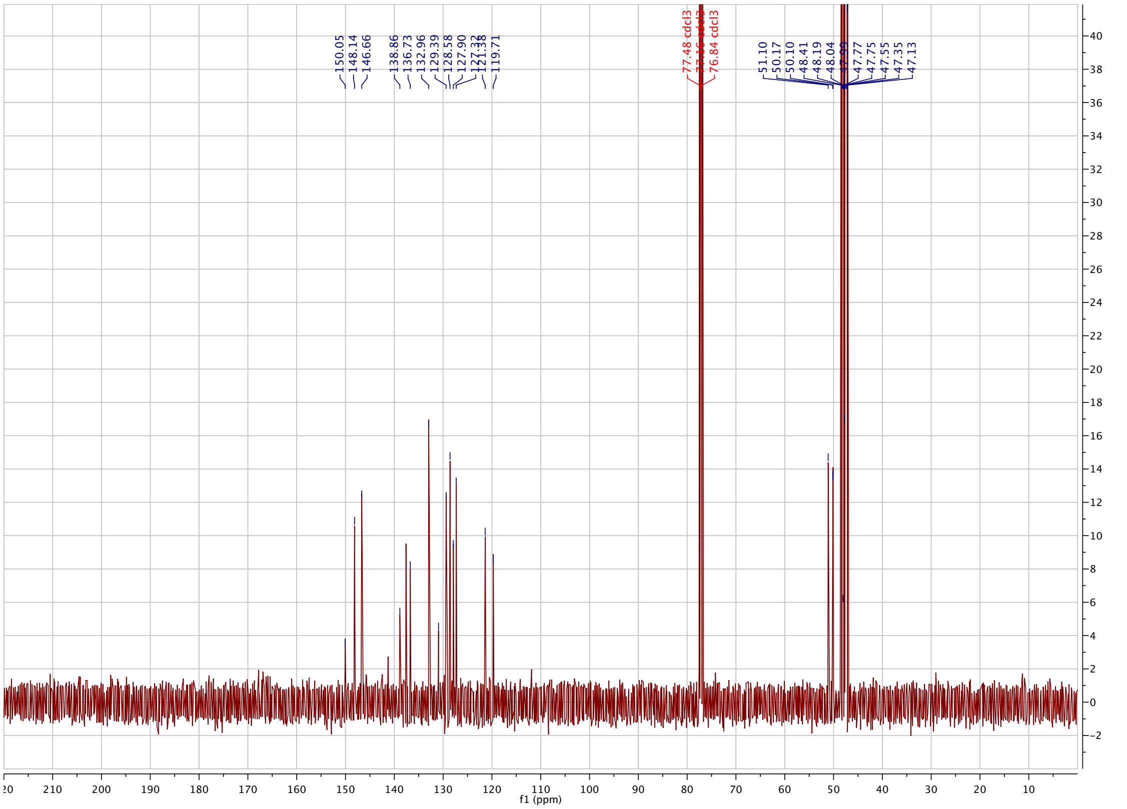

Spectra ^{1}H and ^{13}C of compound 14

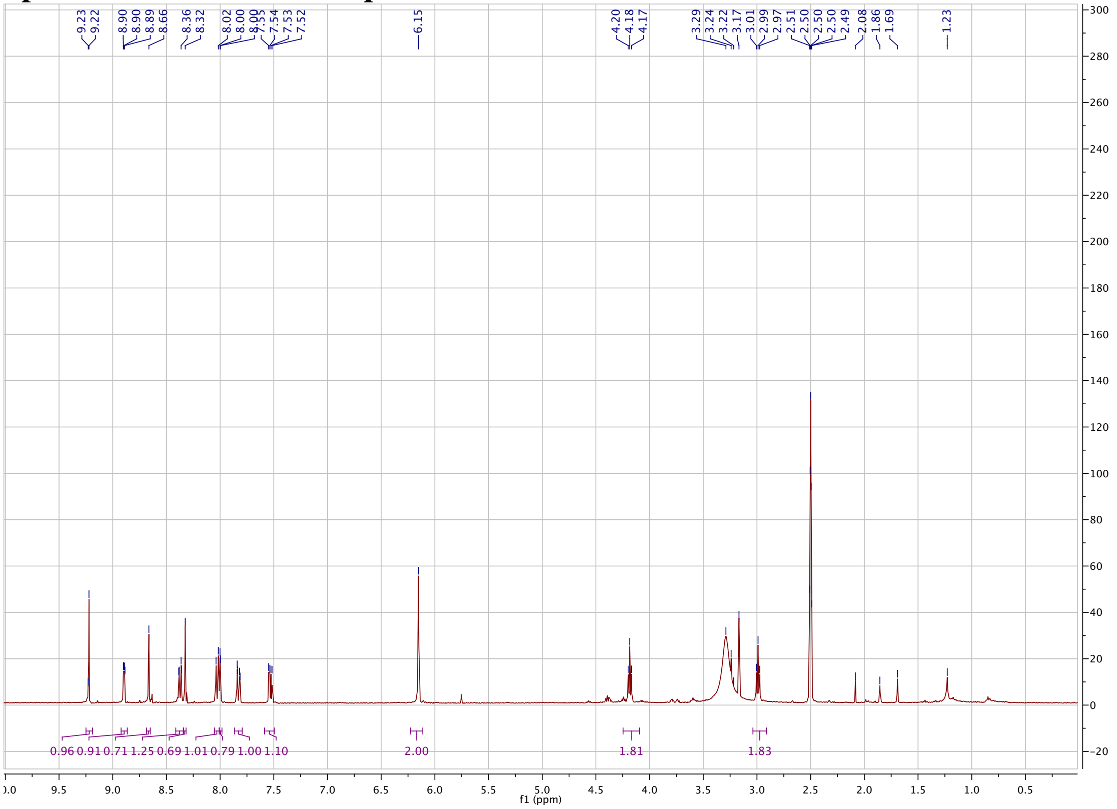

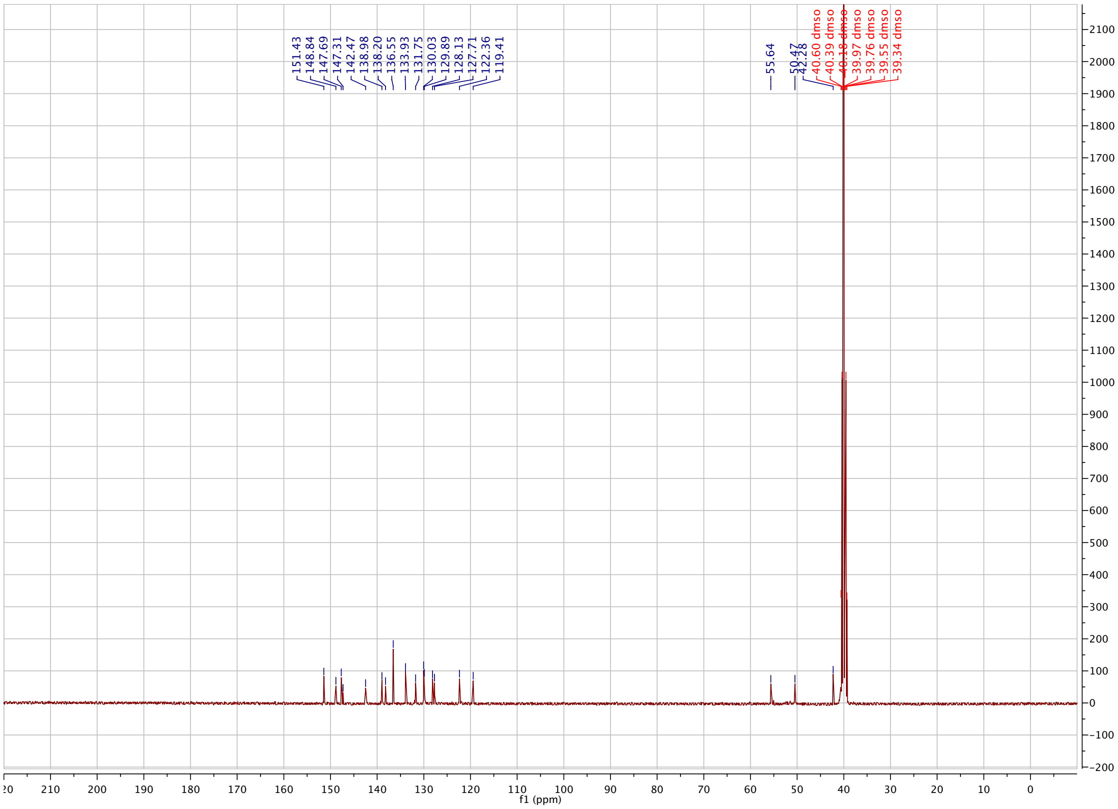

Spectra ^{1}H and ^{13}C of compound 15

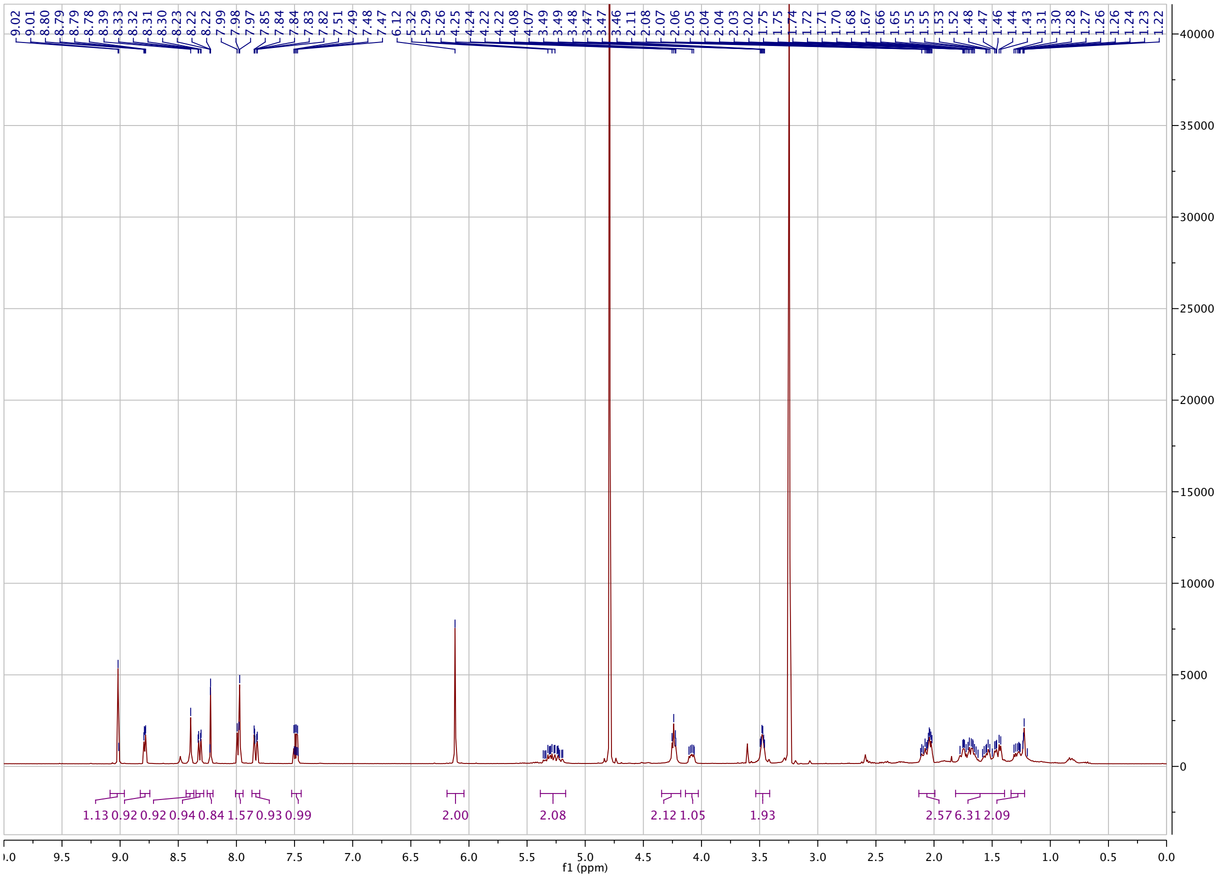

Supplement: File S1 — NMR-spectra of all the products. (PDF) [file pone.0081275.s007.pdf]
